# High-Coverage UHPLC-MS/MS Analysis of 67 Mycotoxins in Plasma for Male Infertility Exposure Studies

**DOI:** 10.3390/toxics12060395

**Published:** 2024-05-28

**Authors:** Xiao Ning, Lulu Wang, Jia-Sheng Wang, Jian Ji, Shaoming Jin, Jiadi Sun, Yongli Ye, Shenghui Mei, Yinzhi Zhang, Jin Cao, Xiulan Sun

**Affiliations:** 1 School of Food Science and Technology, International Joint Laboratory on Food Safety, Synergetic Innovation Center of Food Safety and Quality Control, Jiangnan University, Wuxi 214122, China; nx200730079@163.com (X.N.); jijian@jiangnan.edu.cn (J.J.); sunjiadi@jiangnan.edu.cn (J.S.); yyly0222@163.com (Y.Y.); yinzhizhang@jiangnan.edu.cn (Y.Z.); 2 Key Laboratory of Food Quality and Safety for State Market Regulation, National Institute of Food and Drug Control, Beijing 100050, China; wanglulu199805@163.com (L.W.); yjackyming@126.com (S.J.); 3 Department of Environmental Health Science, College of Public Health, University of Georgia, Athens, GA 30602, USA; jswang@uga.edu; 4 Department of Pharmacy, Beijing Tiantan Hospital, Capital Medical University, Beijing 100070, China; meishenghui1983@126.com; 5 Department of Clinical Pharmacology, School of Pharmaceutical Sciences, Capital Medical University, Beijing 100069, China

**Keywords:** mycotoxin, UHPLC-MS/MS, emerging mycotoxins, male infertility, human biomonitoring

## Abstract

Mycotoxins are a class of exogenous metabolites that are major contributors to foodborne diseases and pose a potential threat to human health. However, little attention has been paid to trace mycotoxin co-exposure situations in vivo. To address this, we devised a novel analytical strategy, both highly sensitive and comprehensive, for quantifying 67 mycotoxins in human plasma samples. This method employs isotope dilution mass spectrometry (IDMS) for approximately 40% of the analytes and utilizes internal standard quantification for the rest. The mycotoxins were classified into three categories according to their physicochemical properties, facilitating the optimization of extraction and detection parameters to improve analytical performance. The lowest limits of detection and quantitation were 0.001–0.5 μg/L and 0.002–1 μg/L, respectively, the intra-day precision ranged from 1.8% to 11.9% RSD, and the intra-day trueness ranged from 82.7–116.6% for all mycotoxins except Ecl, DH-LYS, PCA, and EnA (66.4–129.8%), showing good analytical performance of the method for biomonitoring. A total of 40 mycotoxins (including 24 emerging mycotoxins) were detected in 184 plasma samples (89 from infertile males and 95 from healthy males) using the proposed method, emphasizing the widespread exposure of humans to both traditional and emerging mycotoxins. The most frequently detected mycotoxins were ochratoxin A, ochratoxin B, enniatin B, and citrinin. The incidence of exposure to multiple mycotoxins was significantly higher in infertile males than in healthy subjects, particularly levels of ochratoxin A, ochratoxin B, and citrinin, which were significantly increased. It is necessary to carry out more extensive biological monitoring to provide data support for further study of the relationship between mycotoxins and male infertility.

## 1. Introduction

Mycotoxins are harmful secondary metabolites produced by fungi, commonly found in the environment and food. Strict regulations have been implemented worldwide to establish maximum residue limits for the six types of typical traditional mycotoxins commonly contaminated in food. However, in recent years, some mycotoxins that had received little attention or were newly discovered have been found to have high detection rates in food, such as beauvericin, cyclopiazonic acid, enniatins, and sterigmatocystin [1,2,3,4,5]. These mycotoxins, collectively referred to as emerging mycotoxins, are neither routinely tested nor regulated by legislation [6,7]. Consuming mycotoxin-contaminated foods can lead to acute or chronic diseases. The World Health Organization has identified mycotoxins as major contributors to foodborne illnesses that pose potential threats to human health. Numerous studies have shown that exposure to a mixture of mycotoxins is particularly concerning due to potential synergistic interactions, leading to new and unexpectable effects [8,9]. Therefore, monitoring human exposure to multiple mycotoxins and conducting risk assessments are crucial.

Toxicological studies have indicated that both known and emerging mycotoxins can have negative effects on reproduction [10,11]. Infertility affects about 15% of couples worldwide, with a steady increase [12]. Male factors are responsible for at least 50% of infertility cases [13,14]. The significant decline in sperm quality and human fertility globally has been attributed to various factors [15,16], including the impact of endocrine-disrupting chemicals like mycotoxins [17]. However, the relationship between the development of male infertility and mycotoxin exposure remains unclear due to the lack of reliable data on human internal exposure to mycotoxins. Common biomarkers for mycotoxin exposure involve proteins, DNA adducts, phase I and phase II metabolites, and the parent compounds [18,19]. Only a few methods have effectively detected multiple mycotoxins from different groups in human plasma samples (Table S1). This limited capability has led to underestimations of simultaneous exposure to multiple emerging mycotoxins [20,21,22,23]. Therefore, it is important to establish a detection method to trace foodborne mycotoxins in body fluids that can study the concurrent exposure to mycotoxins and investigate their relationship with male infertility.

Typical assessments of mycotoxin exposure usually involve combining data on their occurrence in food with information about food consumption. However, this approach has certain limitations. For example, the distribution of mycotoxins in food products is not homogeneous, and some mycotoxins are chemically activated by the human body and therefore cannot be detected before consumption. Furthermore, the bioavailability and toxicology of mycotoxins can vary depending on the food treatment and inter-individual differences [24]. Therefore, conducting accurate risk assessments based solely on these data is difficult. Human biomonitoring (HBM) offers a more direct method of investigation by combining the levels of substances in internal body fluids with data on external sources of exposure. HBM provides a more reliable assessment as it correlates mycotoxins with specific disorders rather than focusing solely on food contamination. Ultra-high liquid chromatography–tandem mass spectrometry (UHPLC-MS/MS) is a highly selective and sensitive technology that can be used to analyze multiple chemically diverse mycotoxins from complex biological matrices. Optimizing the extraction and clean-up steps is crucial for implementing this multi-mycotoxin analytical method since mycotoxins have different acid/base properties and cover a wide range of polarities [24,25].

The critical aspect of this work is the rational grouping to achieve the simultaneous analysis of as many components of the same class as possible. During biomonitoring studies, the presence of specific mycotoxins is often unpredictable. The availability of a method capable of precisely quantifying a wide range of commonly occurring mycotoxins in the food supply has significantly lowered the cost of individual sample analysis compared to methods that target a single mycotoxin. It allows for monitoring of large number of samples (hundreds or thousands) to identify sub-populations that may exceed recommended exposure guidelines.

The aim of this study was to develop a rapid, sensitive, accurate, and robust strategy for the quantitative identification of 67 mycotoxins in human plasma, employing a combination of isotope dilution and internal standard quantification through UHPLC/MS/MS. Specifically, isotope dilution mass spectrometry (IDMS) was utilized for approximately 40% of the analytes, mainly for those where reference standards were available, while the remaining were quantified using internal standard methods. This approach allowed us to include both traditional and emerging mycotoxins. The applicability of the improved UHPLC-MS/MS method was then demonstrated by analyzing plasma from 184 males from China, including 89 infertile individuals and 95 fertile individuals. Finally, the study investigated the correlation between male infertility and mycotoxin exposure. To the best of our knowledge, this is the first study in China to evaluate exposure to 67 mycotoxins by analyzing plasma samples from infertile males.

## 2. Materials and Methods

### 2.1. Materials and Equipment

The information about the types, molecular formulas, and structures of 67 mycotoxins obtained from Romer Labs (Tulln, Austria) are shown in Table 1 and Table S2. LC-MS grade acetonitrile (ACN), 99% formic acid (FA), glacial acetic acid (HAc), ammonium acetate (CH_3_COONH_4_), and ammonium formate (NH_4_HCO_2_) were supplied by Fisher Scientific (Waltham, MA, USA). Ultrapure water was generated using a Milli-Q purification system (Millipore, MA, USA). Captiva EMR-Lipid (1 mL) cartridges and Oasis PRiME-HLB (30 mg, 1 mL) cartridges were purchased from Agilent Technologies (Santa Clara, CA, USA) and Waters (Milford, MA, USA), respectively.

Analyses were conducted using a SCIEX ExionLCTM AD liquid chromatography-tandem triple QuadTM 7500 mass spectrometry (AB SCIEX, Framingham, MA, USA) system. Data acquisition and processing were performed using the accompanying software (AB SCIEX, Framingham, MA, USA, version 2.2).

### 2.2. Sampling

This observational study was conducted in the Beijing Obstetrics and Gynecology Hospital, Capital Medical University. Adult males aged 21–49 years who underwent a medical exam or attend an infertility clinic were included in this study. From December 2014 to August 2015, we recruited 184 subjects who met the inclusion and exclusion criteria. The criteria for inclusion in the case (*n* = 89) and the control group (*n* = 95) are detailed in Supplementary Materials. The study was approved by the ethics committee of the Beijing Obstetrics and Gynecology Hospital, Capital Medical University (ethics number 20141201). All participants signed written informed consents. Blood samples were collected after overnight fasting using EDTA vacuum tubes by professional nurses. Plasma samples were separated by centrifugation at 900× *g* for 15 min. Then, the plasma samples were aliquoted and stored at −80 °C in freezers until analysis.

### 2.3. Sample Preparation

Group A. The corresponding 10 μL mixed isotope-labeled internal standard (IS) was added to 0.1 mL of homogenized human plasma samples. After being shaken and mixed, the mixture was incubated for 30 min at 25 °C. Next, 0.4 mL of ACN acidified by 0.1% FA was added to the mixture for protein precipitation, ultrasounded for 20 min, and centrifuged at 9600× *g* for 5 min. Following that, the supernatant was dried under nitrogen at 40 °C and re-dissolved in 0.1 mL of ACN/water (1:9 *v*/*v*). The solution was mixed for 30 s using a vortex and centrifuged at 13,800× *g* for 10 min. The supernatant was then taken for further analysis.

Group B. Initially, 10 μL of mixed isotope-labeled internal standard (IS) was added to 0.1 mL of homogenized human plasma samples. The mixture was vigorously shaken and then incubated at 25 °C for 30 min to ensure thorough integration of the IS. Following incubation, 0.4 mL of ACN/water (84:16, *v*/*v*) was added for protein precipitation. This mixture was subjected to ultrasonication for 20 min and subsequently centrifuged at 9600× *g* for 5 min to separate the supernatant. For purification, the supernatant was processed through Captiva EMR-Lipid cartridges. After the column effluent was collected, 1 mL of ACN/water (4:1, *v*/*v*) was passed through the column to elute any remaining analytes. The eluates were combined, dried under nitrogen at 40 °C, and then reconstituted in 0.1 mL of ACN/water (1:9, *v*/*v*). The final mixture was vortexed for 30 s and centrifuged at 13,800× *g* for 10 min. The clear supernatant was then collected for further analysis.

Group C. The sample preparation process for Group C was similar to that of Group B, with the primary difference being the purification step. Instead of Captiva EMR-Lipid cartridges, PRiME-HLB cartridges were used for purifying the supernatants. After collecting the column effluent, 1 mL of ACN was passed through the column to ensure complete elution. The effluents were then combined and processed in the same subsequent steps as in Group B.

### 2.4. UHPLC-MS/MS Analysis

A Poroshell 120 EC-C18 column (2.1 × 150 mm, 2.7 μm, Agilent, Santa Clara, CA, USA) was selected for chromatographic separation. Detailed information on the applied mobile phase gradients and optimization information about other chromatographic conditions can be found in the Supplementary Materials, including the MS/MS, scheduled multiple reaction monitoring (SMRM), and ion source parameters. Table S3 summarizes the MS parameters of 67 mycotoxins and 27 labeled IS.

### 2.5. Method Validation

The validation of the method was conducted in compliance with the established guidelines [26,27,28], and the specific indicators and methodology were as follows.

#### 2.5.1. Selectivity and Limit of Quantitation (LOQ)

The selectivity determination of methodological refer to [29]. The LOQ was determined as the lowest concentration that satisfies a minimum signal-to-noise ratio of 10.

#### 2.5.2. Carry-Over Effect and Linearity

Determination of carry-over effects and acceptable levels refer to the article of Ediage et al. [30]. Matrix-matched calibration curves were prepared daily for the quantification of mycotoxins in plasma, ranging from the LOQ to 100 times the LOQ, with a labeled IS concentration set at 20 times the LOQ. Blank plasma was spiked with a combined standard of mycotoxins to achieve a concentration of 100 times the LOQ for each mycotoxin. The preparation of calibration curve samples, HPLC-MS/MS analysis, and calculations were based on the method proposed by Slobodchikova et al. [31]. The selection of the corresponding IS for each analyte is clearly presented in Table S3.

#### 2.5.3. Trueness and Precision

Accuracy encompasses both trueness and precision, reflecting the influence of both systematic and random errors. The trueness of the method was reflected in the average recovery of 67 analytes at three spiked levels in the blank male plasma. Precision was determined by analyzing the spiked samples for six replicates within a single day (intra-day) and over three consecutive days (inter-day) and expressed in terms of the RSD. The bias and RSD of validation samples should be within ±20% and less than 15%, respectively.

#### 2.5.4. Recovery and Matrix Effect

The quality control (QC) samples were randomly prepared with plasma from 12 control groups and stored at −80 °C before use. Three batches of validation samples at three levels were prepared according to Yang et al. [32]. Briefly, 67 mycotoxins and IS were added in mixed plasma from 12 individuals in control groups (set as A), in water (set as B), and post-protein precipitated in mixed plasma from 12 individuals in control groups (set as C). The three batches were then treated and analyzed based on the optimal methods. The recovery rate was calculated as the percentage of the ratio of A to C, and the matrix effect was calculated as the percentage of the ratio of C to B.

#### 2.5.5. Stability

Stability studies include long-term stability studies and short-term stability studies. In the short-term stability test, the recovery of validation samples at 2-fold and 100-fold LOQ were measured under different time-temperature combination conditions (in plasma: 4 °C or 25 °C for 3 h, 6 h, 1 day, 3 days, and 5 days and three freeze-thaw cycles from −80 °C to 25 °C; post treatment: 4 °C for 120 h). The samples were stored at −80 °C for two months in the long-term stability study, otherwise under the same conditions as in the short-term study.

### 2.6. Statistical Analysis

Categorical variables were presented as frequencies and percentages. The Shapiro–Wilk test was used to examine the normality of the continuous variables. Each mycotoxin concentration exhibited a skewed distribution; therefore, the median and interquartile range were used to describe the mycotoxin concentrations. Chi-squared or Fisher’s exact tests were applied to compare the differences in mycotoxin detection rates between the case and control groups. The concentrations of mycotoxins with positive detection rates of more than 70% were compared, and the concentrations below the detection levels were imputed using 50% of the LOD. The Spearman correlation analysis was used to analyze the correlations between different mycotoxins. All statistical analyses were performed using StataMP version 16.0 and R 4.2.2.

## 3. Results and Discussion

### 3.1. Optimization of Mobile Phase

**Figure 1:**
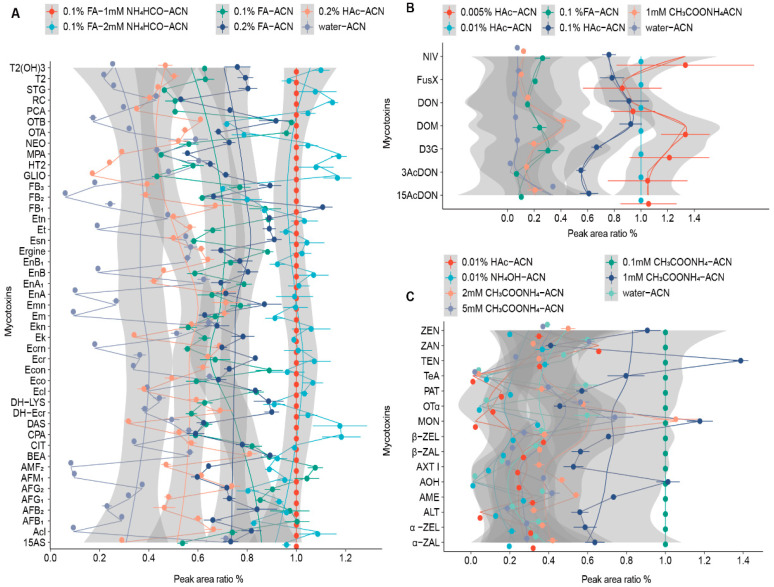
Effects of different mobile phase conditions on peak areas. (**A**) 45 mycotoxins of group A. (**B**) 15 mycotoxins of group B. (**C**) 7 mycotoxins of group C (*n* = 3). The concentrations of mycotoxins in the samples were as follows: Acl, Ecl, Em, Esn, CPA, STG, EnA1, EnB, EnB1, Ergine, Econ, Etn, OTA, OTB, AFM1, and AFM2: 0.5 ng/mL. Ek, Ekn, Emn, MPA, NEO, RC, T2, DH-Ecr, EnA, and BEA: 1 ng/mL. 15AS, AFB1, AFB2, AFG1, AFG2, Ecr, Ecrn, Eco, T2(OH)_3_, AME, ZEN, AXT I, OTα, DAS, DH-LYS, Et, and FB3: 5 ng/mL. ALT, TeA, FB1, FB2, HT2, PCA, CIT, AOH, TEN, α-ZAL, β-ZAL, α-ZEL, β-ZAL, ZAN, and D3G: 10 ng/mL. 3AcDON, FusX, NIV, GLIO, DON, DOM, and MON: 50 ng/mL. PAT, and 15AcDON: 100 ng/mL.

During the quantitative analysis of compounds in complex biological matrices, efficient chromatographic separation is important to avoid ionization interference of the MS source and to enhance the sensitivity and accuracy of the entire analysis. Thus, effects of mobile phase composition on the chromatographic performance were investigated. Figure 1 shows the ratio of the peak area of each analyte under process conditions to the peak area under the final optimization conditions. As shown in Figure 1A, an acidic mobile phase generated abundant hydrogen ions, which assisted the positive ionization and enhanced the sensitivity of detection of most analytes in group A, such as OTs, FBs, and AFs. The FA (green and blue dot) was more effective compared to the weaker acid HAc (orange dot). An increase in FA concentration from 0.1% to 0.2% improved the peak shapes and sensitivity of detection of FBs and some emerging mycotoxins, including ergot alkaloids, MPA, STG, and GLIO. However, this change suppressed the signal intensity of some important mycotoxins, including AFs and OTs. Therefore, the optimal concentration of FA was set as 0.1%. Notably, this mobile phase composition did not lead to sufficient signal intensity for the detection of T2, HT2, and some emerging mycotoxins, such as BEA, enniatins, DAS, NEO, and 15AS.

When NH_4_HCO_2_ was included in the mobile phase, the signal intensities of the NH_4_^+^ adducts produced were higher than those of the H^+^ adducts. However, higher concentrations of NH_4_HCO_2_ inhibited the ionization of most regulated mycotoxins, such as AFs, OTs, and FBs, which is consistent with the results of Qiu et al. [33]. Moreover, all mycotoxins exhibited maximum signal intensities when mobile phase (A) consisted of 1 mM NH_4_HCO_2_ with 0.1% FA in aqueous solution and mobile phase (B) was ACN. Although CPA analysis under neutral conditions led to tailing peaks, sharp peaks and good separations were obtained using the optimized acidic mobile phase. However, the signal intensities of most compounds in group B were suppressed in acid or alkaline media. Previous studies reported that the addition of CH_3_COONH_4_ in the mobile phase could improve the sensitivity of detection of ZEN and its derivatives and peak shapes of several Alternaria toxins [34,35]. Results showed that the combination of 0.1 mM CH_3_COONH_4_ (A) and ACN (B) markedly enhanced the signal intensities of most target components and eliminated the tailing peaks of TeA and PAT (Figure 1C). Thus, 0.1 mM CH_3_COONH_4_ and ACN were selected as the mobile phase with the best-balanced performance.

Regarding group C compounds, when HAc was used instead of FA at the same concentration of 0.1%, the intensity of the analytes increased from 1.7-fold (for D3G) to 6.0-fold (for 3AcDON) (Figure 1B). The [M+CH_3_COO]^−^ provided by HAc greatly enhanced the ionization of the analytes. Upon reducing the concentration of HAc from 0.1% to 0.01%, the signal intensity of 3AcDON increased by 1.8-fold. A further decrease from 0.01% to 0.005% improved all signal intensities, except for DON and FusX. However, the use of 0.005% HAc resulted in poor precision for all analytes, with the RSD of the peak area for the tested mycotoxins (*n* = 3) ranging between 11% and 32%. On the other hand, the RSD was between 1% and 4% when 0.01% HAc was used, and the signal intensities of all analytes improved, while the peaks of NIV and D3G became sharper. Therefore, a 0.01% HAc aqueous solution was chosen as mobile phase (A), and ACN was used as mobile phase (B) for the group C compounds.

### 3.2. Optimization of Ion Source Parameters

To further enhance performance, we manually optimized the key parameters that impact the ionization procedure, such as ionization mode and the curtain gas, in a stepwise manner. We conducted tests on the ion spray voltage (ISV) ranging from 1400 to 2600 V, the source temperature ranging from 350 to 650 °C, and the pressures of the nebulizer gas (gas1) and auxiliary gas (gas2) ranging from 30 to 80 ps. These ion source parameters had varying effects on the peak areas of the 67 mycotoxins.

**Figure 2:**
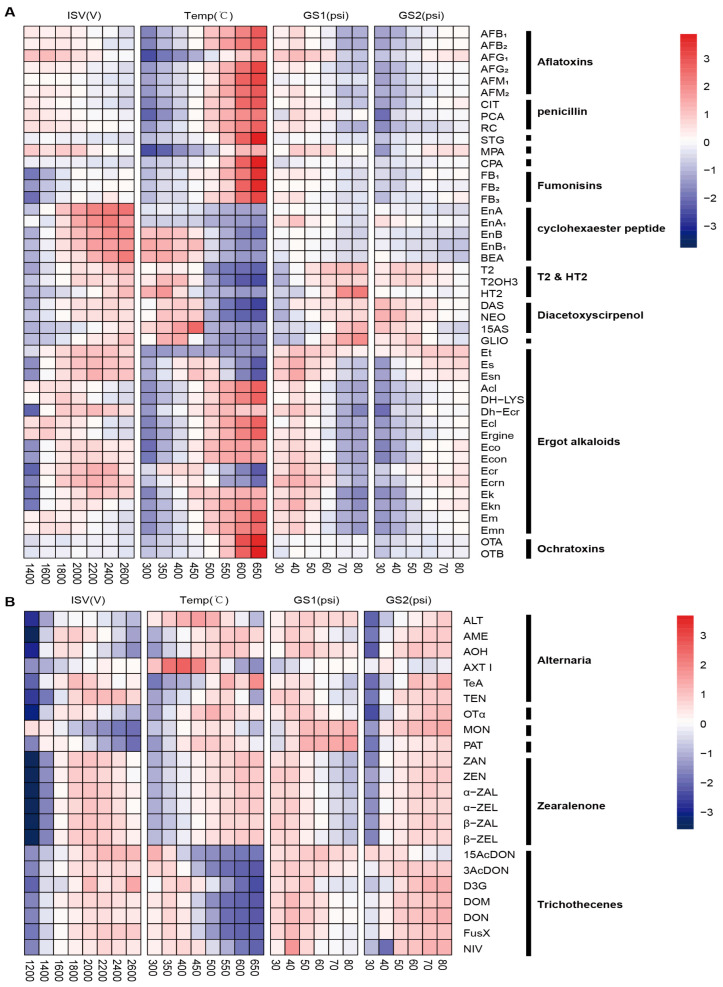
Evaluation of the effects of ion source parameters on detection sensitivity. The values were presented as the ratio of the peak area under the noted conditions to the peak area under initial conditions. (**A**) Group A analytes. (**B**) group B analytes. (*n* = 3). The concentration of each mycotoxin is described in the legend of Figure 1.

As shown in Figure 2A, the peak areas of FBs, T2, HT2, T2(OH)_3_, cyclohexaester peptides (BEA and enniatins), and diacetoxyscirpenols (DAS, NEO, and 15AS) substantially increased with each increment of ISV. Conversely, the peak areas of AFs, STG, CPA, MPA, CIT, PCA, and RC slightly decreased. Regarding OTs, ergot alkaloids, and their respective inin-epimers, their optimal ISV value was achieved at 2000 V, with the peak areas diminishing at both higher and lower ISV values. Hence, 2000 V was chosen as the ISV for analyzing group A mycotoxins. While most analytes exhibited an increase in signal intensity with rising temperature, T2, HT2, T2(OH)_3_, cyclohexaester peptides, diacetoxyscirpenols, and GLIO displayed a significant decrease when the temperature surpassed 450 °C. Therefore, 450 °C was selected as the optimum temperature.

The peak areas of T2, HT2, T2(OH)_3_, diacetoxyscirpenols, and GLIO were directly proportional to the pressure of gas1, whereas other analytes showed optimal sensitivity when the pressure of gas1 reached 40 psi. As the pressure of gas2 increased, the peak areas of most analytes also increased, except for cyclohexaester peptides and diacetoxyscirpenols, which showed a decrease in sensitivity. To cater to the requirements of most mycotoxins, the pressure of gas1 was maintained at 40 psi, while gas2 was kept at 80 psi. These conditions provided adequate sensitivity of detection for all analytes. Concerning group B mycotoxins, increasing the ISV value resulted in a uniform increase and subsequent decrease in the peak area of all analytes (Figure 2B). The optimum performance was achieved at an ISV of −1800 V at 500 °C, with gas1 and gas2 pressures set at 40 and 80 psi, respectively. For compounds in group C, the relationships between the ion source parameters and the response values of the analytes were similar to those in group B. Most compounds in group C exhibited a slight increase in peak areas with increasing gas2 pressure, except for 15AcDON, which saw a decrease in the peak area (Figure 2B). Therefore, a moderate value of 50 psi was used for the pressure of gas2.

### 3.3. Optimization of the Pre-Treatment Methods

Enhanced Matrix Removal-Lipid (EMR-Lipid) cartridges and PRiME Hydrophile-Lipophile Balance (PRiME-HLB) cartridges are two novel polymer-based sorbent technologies that promise highly selective removal of phospholipids and proteins from complex matrices [36]. The extraction solvent was optimized by considering the physical interactions of mycotoxins with lipids and/or proteins in serum, as well as the wide range of log *p* values (ranging from −1.9 to 4.74) of the 67 mycotoxins. Additionally, a SPE (PRiME-HLB or EMR-Lipid) clean-up step was included to assess the efficiency of the combined extraction steps in inhibiting the matrix interference and achieving satisfactory recovery for all mycotoxins.

**Figure 3:**
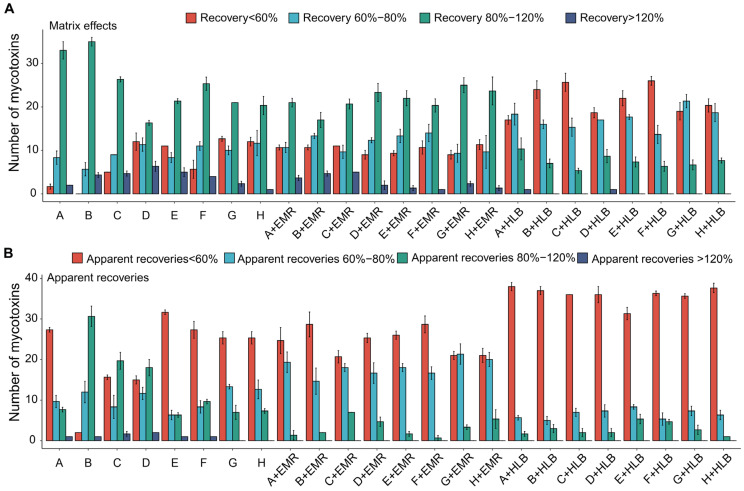
Assessment of the effects of solid-phase extraction purification on the matrix effects (**A**) and apparent recovery values (**B**) of 45 mycotoxins in group A. The abbreviations in the illustration are as follows: A: ACN; B: 0.1% FA ACN; C: 0.5% FA ACN; D: 1% FA ACN; E: water-ACN (16:84, *v*/*v*); F: 0.1% FA ACN (16:84, *v*/*v*); G: 0.5% FA ACN (16:84, *v*/*v*); H: 1% FA ACN (16:84, *v*/*v*). The concentration of each mycotoxin is described in the legend of Figure 1.

Concerning group A compounds, complex acidic analytes such as FBs and OTs necessitated a higher water content or a lower pH for efficient extraction. However, a higher water content impeded the extraction of other analytes, leading to the conclusion that pH adjustment is a preferable approach for optimizing extraction [37]. The use of FA-acidified ACN as an extraction solvent significantly improved the recoveries of strongly polar analytes such as FBs and OTA, which qualitatively matched the findings reported by Arce-López et al. [36]. Nonetheless, FA concentrations exceeding 0.1% decreased the extraction efficiency. Notably, mycotoxins such as RC, STG, DAS, Ecl, Ergine, and PCA exhibited recoveries below 80% or above 120%. The M_E_ evaluation of these mycotoxins indicated that these variations in recovery were a result of ionization suppression or enhancement, rather than poor extraction. Satisfactory recoveries for these analytes were obtained after performing IS corrections, underscoring the necessity for quantitation using IS. Figure 3 presents a comparison of the R_A_ and M_E_ values obtained from different sample preparation protocols for 45 group A mycotoxins.

Regarding the analytes monitored in the negative ion mode for groups B and C, it was found that an acidic extraction solvent was not suitable, and the lack of FA resulted in substantial ME. To address this issue, an SPE cartridge was used to filter out impurities in plasma, which improved the ionization of target mycotoxins by reducing the matrix effect. However, a single column was not appropriate for both groups. The unsatisfactory recovery rates (<70%) of ZAN (28–63%), AOH (10–33%), TeA (6–57%), MON (13–45%), and PAT (8–61%) using PRiME-HLB (HLB) could be attributed to factors such as non-specific adsorption and the limited retention capacity of reversed-phase hydrophobic sorbents for highly polar analytes. In Figure S1, it can be observed that when ACN/water (84/16, *v*/*v*) was used as the extraction solvent in combination with an EMR-Lipid SPE clean-up step, the recoveries of all compounds in group B were above 70% before IS correction, except for AOH (52.4%) and PAT (48.3%). Similarly, when ACN was used as an extraction solvent followed by purification with an HLB cartridge, the recoveries of DON and its derivatives were higher than 70% except for NIV (42.4%). These results indicate that most mycotoxins exhibit poor recoveries due to their suppression of ionization, except for AOH, PAT, and NIV, which had low extraction efficiency. Additionally, no interfering peaks were detected at retention times and *m*/*z* channels that were similar to the mycotoxins (Figure S2), demonstrating the selectivity of the method and absence of interference from endogenous substances.

### 3.4. Methodology Validation

#### 3.4.1. LOQ and Selectivity

Supplementary Figure S2 shows the typical UHPLC-MS/MS chromatograms of the validation samples prepared by spiking blank plasma at LOQ levels. The presence of a labeled IS did not impart any measurable influence on the quantification of all analytes. Despite the observation of some interfering peaks at the elution time of analytes, their responses were significantly lower, and they accounted for far less than 20% of the response shown at the LOQ level for each analyte. The labeled IS did not have any significant effect on the quantification of all analytes. The signal-to-noise ratios of LOQ samples were more than 11.3. The LODs ranged between 0.001 μg/L (Acl, Ecl, Em, Esn, EnA1, EnB, EnB1, CPA, and STG) and 0.5 μg/L (PAT), with corresponding LOQs ranging between 0.002 and 1 μg/L. Compared to previously reported methods of mycotoxin analysis, our method allows for the detection of the largest number of analytes while also offering the highest reported sensitivity. Notably, the LODs of FusX, OTB, NIV, T2, HT2, NEO, ZAN, and STG attained with the proposed method were lower compared to those attained with a single method that employed a clean-up step using EMR-Lipid [34]. This finding demonstrates the need to design sample preparation methods that consider the physicochemical properties of mycotoxins.

#### 3.4.2. Carry-Over Effect and Linearity

There was no carry-over effect for all analytes. Satisfactory linearities were obtained for all mycotoxins (R^2^ range of 0.9902–0.9999).

#### 3.4.3. Trueness and Precision

As shown as Table S4, at three validation levels, the mean intra-day trueness ranged from 82.7% to 116.6%, and intra-day precision ranged from 1.8% to 11.9% RSD, except for Ecl (66.4% to 78.7%), DH-LYS (75.2% to 78.2%), PCA (72.5% to 77.4%), and EnA (117.1% to 129.8%). The analysis of 63 out of 67 mycotoxins met the trueness requirement and precision. The inter-day trueness ranged between 80.2–117.7%, while the precision ranged from 3.1–13.8% RSD. The results indicate that this method effectively analyzes trace concentrations of mycotoxins.

#### 3.4.4. Recovery and Matrix Effect

In Figure S1, when ACN/water (84/16, *v*/*v*) was used as the extraction solvent in combination with an EMR-Lipid SPE clean-up step, the recoveries of all compounds in group B were greater than 70% before IS correction, except for AOH (52.4%) and PAT (48.3%). Similarly, when ACN was used as an extraction solvent followed by purification with an HLB cartridge, the recoveries of DON and its derivatives in group C were higher than 70%, except for NIV (42.4%). These results suggest that most mycotoxins show poor recoveries due to their suppression of ionization, except for the low extraction efficiency of AOH, PAT, and NIV. Due to the complexity of the matrix, the matrix effect values ranged between 62.5% and 155.6%, while recovery values ranged between 59.6% and 146.4%, indicating the need for IS compensation for accurate mycotoxins analysis. For the analytes without commercially available standards, IS that showed comparable recovery values were chosen as reference IS.

#### 3.4.5. Stability

Data obtained from the short-term stability studies revealed an almost insignificant degradation (<10%) of the different analytes at the different time-temperature combinations, except for CIT, AFM_1_, AFB_1_, AFG_1_, and AFG_2_ for which the two-fold LOQ spiked analyte concentrations were unstable after 6 h at 25 °C, and 10% to 20% of the initially spiked analyte concentrations was lost (Figure S3). This result is in agreement with the study by Ediage et al. [30]. The 120 h stability shows that very long analytical batches, suitable for exposure monitoring studies, can be accommodated using the current method.

Further short-term stability investigations carried out at temperatures of 4 °C and 25 °C for durations of 1, 3, and 5 days revealed notable losses (Table S5). Specifically, samples stored at 25 °C for 5 days exhibited a reduction of at least 20% in their initial analyte concentrations. The analytes that underwent the most degradation were CIT, T2 toxins, HT2, AFM1, AFB1, AFG1, AFG2, DON, and DOM (Figure S3). Remarkably, less than 30% of the initial T2 toxin and AFG1 concentrations were retained after 5 days, regardless of the storage temperature. In contrast, the non-polar analytes within the group (including ZAN, ZEN, OTA, and their derivatives) demonstrated minimal degradation compared to the polar analytes such as DON and its derivatives, across all time-temperature combinations.

In response to concerns regarding the potential effects of long-term storage on our samples, it is crucial to note that our methodology required plasma samples to be thawed on ice and promptly processed to mitigate degradation. For the long-term stability test, minimal degradation (<5%) was observed in the various analytes after 2 months of storage at −80 °C. This minimal change was corroborated by applying identical collection and storage protocols to both control and case groups in the latter part of our study. This approach was designed to ensure that any potential degradation effects were systematically controlled and consistent across all samples, thus providing a reliable basis for our comparative analysis. This synchronization in handling and storage mitigates the variability that might otherwise arise from differential treatment of samples, allowing us to draw more accurate inferences about the correlation between mycotoxin exposure and male infertility.

### 3.5. Detection of Mycotoxin Levels in Plasma Samples

Mycotoxins are lipophilic and can attach to proteins in plasma, allowing them to persist in organisms for long periods of time after chronic exposure [38]. The presence of mycotoxins in urine is usually a sign of recent ingestion, whereas the presence of mycotoxins in plasma is more closely associated with long-term exposure [39]. Therefore, compared to urine, plasma samples are more suitable for biological monitoring, permitting the generation of more comprehensive and reliable data for the creation of risk assessments. The UPLC-MS/MS chromatograms of blank plasma samples and plasma matrices spiked with standards representing the 67 mycotoxins are presented in Figure S2. The mycotoxin contents in plasma from 184 human male subjects are shown in Table S6. Among the 67 analyzed mycotoxins, 16 of the 28 traditional mycotoxins and 24 of the 39 emerging mycotoxins were detected in the plasma samples. The most prevalent mycotoxins included both traditional mycotoxins (OTB at 100% and OTA at 98.4%) and emerging mycotoxins (CIT at 79.3%, EnB at 77.2%, BEA at 68.5%, and CPA at 44.6%), suggesting the widespread exposure of both traditional and emerging mycotoxins in male plasma.

The prevalence of these analytes in various foods has become increasingly well established. Globally, OTA contamination has been frequently documented in raw agricultural products, including grain, coffee, peas, and meat [40,41]. In 2017, a report on OTA contamination detection in rice samples from various regions in Africa showed that out of 4000 samples, the OTA content in raw and processed grains exceeded 38% and 29%, respectively, with the highest level of OTA content being 1164 μg/kg [42]. A study performed in Tehran, Iran, reported similar results, with 69 out of 100 rice samples containing OTA. In previous studies, CIT has been detected in various plant-based food commodities, particularly in cereals, fruit, and vegetables [43]. Qiu et al. revealed that more than 80% of the 13 types of food samples in the 6th China Total Dietary Study were contaminated with mycotoxins [33]. In our results, the most frequently detected mycotoxins were BEA and EnB.

**Figure 4:**
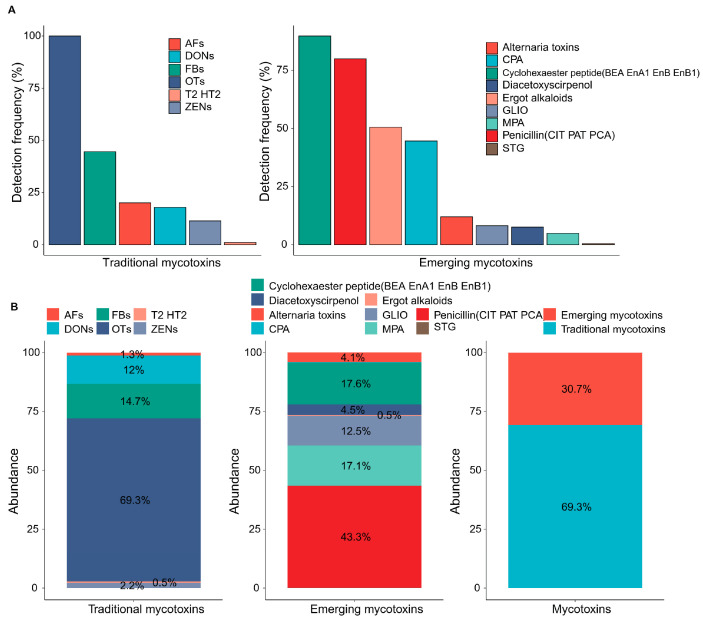
Prevalence and composition profiles of mycotoxins in plasma samples collected from 184 Chinese males. (**A**) Detection frequency of both traditional and emerging mycotoxins. (**B**) Abundance levels of traditional and emerging mycotoxins.

In Figure 4, it is observed that OTs, FBs, and DONs were identified as the most abundant traditional mycotoxins, with an average combined contribution ratio from 12.0% to 69.2% of the total of traditional mycotoxins. Additionally, penicillins (CIT, PAT, and PCA), cyclohexaester peptides (EnB, BEA, EnB_1_, and EnA_1_), MPA, and GLIO were the predominant emerging mycotoxins, with a combined average contribution ratio of 12.5–43.3%. Apart from these mycotoxins, which consistently occurred at relatively high concentrations, the remaining detectable mycotoxins were present in low concentrations, collectively contributing to less than 6% of the total mycotoxin burden. Despite their low concentrations, some mycotoxins, including two emerging mycotoxins (ergot alkaloids at 50.5% and CPA at 44.6%), were detected more frequently, suggesting their widespread occurrence in common foods. The high detection rate of ergot alkaloids was mainly derived from Esn (23.9%), Acl (14.1%), and DH-LYS (13.6%), whereas CPA was detected individually.

### 3.6. Analysis of Major Co-Exposure Mycotoxins

Figure S4 shows representative MRM chromatograms of the positive samples. The results indicate that all participants were exposed to a minimum of two different mycotoxins. Interestingly, the plasma from one infertile participant was found to contain at least 14 mycotoxins. The established mycotoxins OTA, AFB_1_, FB_1_, and DON are known to induce negative reproductive effects, including reductions in the weight of reproductive organs, daily sperm production, epididymal sperm count, and the numbers of viable and motile sperm [44,45,46,47]. The emerging mycotoxins CIT and CPA have exhibited similar effects. CPA has been shown to decrease the sperm quality and rates of in vitro fertilization success in mice, and it has been found to induce p53-dependent apoptosis in the testis of mice [48,49,50]. Among all the detected mycotoxins, OTA, OTB, EnB, and CIT exhibited detection frequencies higher than 70%.

**Figure 5:**
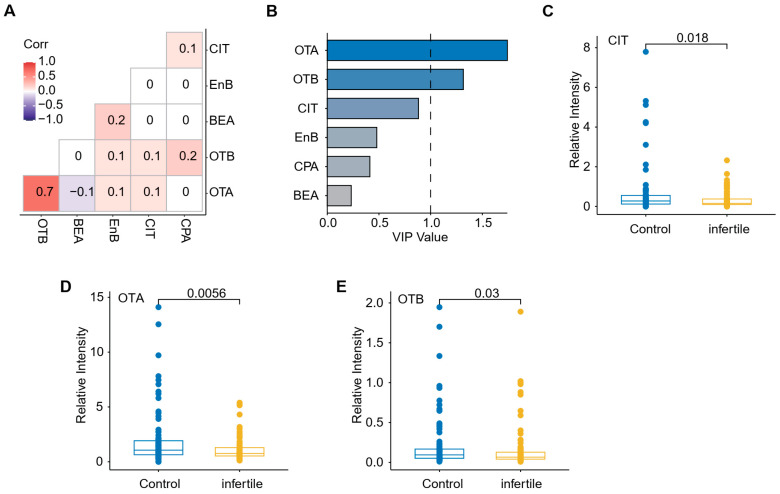
Multivariate statistical analysis of mycotoxin levels in plasma samples. (**A**) Heatmaps for Spearman’s correlation coefficients between concentrations of metabolites in plasma samples. (**B**) VIP bar plot of mycotoxins based on the OPLS-DA model. (**C**–**E**) Boxplot of the plasma levels of mycotoxins (CIT, OTA, and OTB) with a high (>70%) incidence between normal and infertile male plasma.

As shown in Figure 5A, positive concentration correlations were observed between OTA and OTB (r = 0.72, *p* < 0.01), OTA and EnB (r = 0.36, *p* < 0.01), OTA and CIT (r = 0.21, *p* < 0.01), OTB and CIT (r = 0.22, *p* < 0.01), OTB and EnB (r = 0.33, *p* < 0.01), and EnB and CIT (r = 0.17, *p* < 0.05), indicating that these mycotoxins co-migrate in some common food sources. These results were consistent with Gupta et al., who determined that *Penicillium* and *Aspergillus* spp. can produce both OTs and CIT. This co-production means that co-exposure to these mycotoxins is common in foods. Out of 250 grain samples, five contained OTA (147 ± 7.9 μg/kg), CIT (49 ± 1.9 μg/kg), and OTB (1.2 ± 0.7 μg/kg) [48]. Previous studies have also reported that fungi of the genus *Penicillium* can degrade CIT, leading to the production of OTA. Consequently, the European Union and other interntional organizations have warned that the chronic toxicity of such emerging toxins and the synergistic toxicity of co-exposure with traditional toxins may pose a threat to human health.

The mean OTA concentration (0.904 μg/L) and range (0.117–14.103 μg/L) observed in infertile males in this study were similar to those reported in a survey on rural residents aged 18 to 66 years in China and to those observed in ill children in Spain [36,51]. OTα, a metabolite of OTA, was not detected in plasma; the same results were reported in studies of Chinese, German, and Belgian populations [24,51,52]. This is not surprising because OTα is mainly excreted in urine as glucuronide or sulfate conjugates [49,50]. Furthermore, little attention has been paid to OTB exposure, mainly because previous studies have reported a relatively low incidence of 11.4% [37]. In contrast, our results suggest that the incidence of exposure to OTB is 100%, with our method exhibiting an LOD for OTB detection that is approximately 200-fold lower compared to that of the method used by López et al. [37]. It is possible that the relative insensitivity of their method resulted in a high number of false negative results in Spanish children. It should be noted that the mean concentrations obtained in the two studies were similar, 0.22 and 0.57 μg/L, respectively.

The incidences of DON and its derivatives were low in plasma samples, except for FusX, even though their LODs were improved by choosing [M+CH _3_COO]^−^ as the precursor ion. This result is consistent with previous studies on German subjects, in which DON and especially its phase II metabolites were often detectable in urine [53,54], suggesting a rapid excretion of these compounds. To our knowledge, our study is the first to report on plasma exposure to FusX. FusX was detectable because the LOD of our method (0.05 μg/L) is substantially lower than that of the method described by Arce-López et al. (1.95 μg/L) [37]. In addition, it may be that FusX is either produced internally from DON or obtained directly from food or the environment, as it readily accumulates in the plasma and is excreted from the body relatively slowly.

Several HBM studies have revealed a high incidence of CIT in plasma. For example, a study on plasma samples from 104 young adult patients in Bangladesh revealed an incidence of 90%, with a mean plasma concentration of 0.22 µg/L [55]. Similarly, a study in the Czech Republic showed that 100% of subjects with renal tumors were CIT-positive, with a mean concentration of 0.061 µg/L. In a study on subjects from Tunisia, the prevalence was slightly lower at 36%, whereas in our study, the mean concentration was higher at 0.49 μg/L. Our study aligns more closely with a high prevalence of CIT exposure [56]. The high incidence of EnB observed in our study was also consistent with a study on healthy German volunteers [52]. Thus, the present study confirms the emergence of CIT and EnB as critical toxins in plasma and provides novel information on other emerging mycotoxins such as BEA and CPA.

### 3.7. Analysis of Potential Correlation between Mycotoxin Exposure and Infertility

Co-exposure to multiple mycotoxins was frequent among both control and infertile subjects. The most common result was co-exposure to 7–14 mycotoxins. Notably, this finding occurred more frequently among individuals in the infertility group (60%) compared to those in the control group (44%), indicating a strong correlation between multi-exposure and infertility. According to the variable importance of the projection values (VIP) bar plot of mycotoxins selected based on the orthogonal partial least square discriminant analysis (OPLS-DA) model, OTA, OTB and CIT exposure contributed the most to infertility outcome (Figure 5B). The prevalence levels of FusX, ergot alkaloids, Esn, and MPA in the infertility group (19%, 59%, 34%, and 9%, respectively) were notably elevated compared to those observed in the control group (8%, 43%, 15%, and 1%, respectively; *p* < 0.05). However, as these mycotoxins were monitored in a human biological matrix for the first time, the relationship between their occurrence or levels and specific diseases has not been well-studied. It is worth noting that the rate of detection of CPA in infertile male samples (47.2%) was higher than in fertile male samples (42.1%). Additionally, as mentioned above, several studies have demonstrated the harmful effects of CPA on spermatogenesis. Therefore, further studies on the dose-effect relationship of CPA exposure and the pathogenesis of male infertility are warranted.

The Mann–Whitney U test was used to conduct inter-group comparisons of the plasma levels of mycotoxins that were found in more than 70% of the samples (Figure 5B–D). The median values of OTB, OTA, and CIT levels in the infertility group (0.095, 1.070, and 0.329 μg/L, respectively) were higher than those in the control group (0.066, 0.760, and 0.198 μg/L, respectively). All these three mycotoxins showed the highest levels in samples from the infertility group. When the concentrations were analyzed as continuous variables, significant differences were observed between the two groups (all *p* < 0.05). Interestingly, OTB, OTA, and CIT, which were elevated in infertile male subjects, are known to induce reproductive and significant renal toxicities. They have been reported as the cause of kidney diseases in humans and animal models [57,58,59,60]. The apparent toxicity of these mycotoxins, coupled with their high prevalence and concentrations in infertile male subjects, necessitates further research on the toxicity of OTs and CIT alone and in combination in infertile men. As for EnB, another mycotoxin with a high incidence, measured concentrations ranged between 0.002 and 0.037 μg/L in the infertility group and 0.002 and 0.147 μg/L in the control group. No statistical difference was observed between the two groups, suggesting that EnB does not significantly contribute to male infertility.

## 4. Conclusions

This study has successfully developed a comprehensive UHPLC-MS/MS method for the quantitative analysis of 67 mycotoxins in plasma, showcasing its sensitivity, accuracy, and robustness. Our findings underscore the significance of investigating the link between mycotoxin exposure and male infertility, particularly focusing on specific reproductive toxins that could impair reproductive health. Despite the potential association suggested by our results, establishing a definitive connection requires more conclusive evidence.

The role of environmental factors in male fertility is critical, as adverse conditions can significantly impact reproductive capabilities [61]. It is essential to acknowledge that male infertility likely results from the complex interplay of multiple environmental factors, rather than mycotoxins alone. Consequently, further research is needed to validate our findings, understand the underlying mechanisms, and assess the broader impact of environmental toxins on fertility. Expanding the scope of our study to include diverse sample types and larger populations from various geographic regions will help provide a more comprehensive understanding of these interactions and support more definitive conclusions.

## Figures and Tables

**Table 1:** Group information for 67 mycotoxins and based on the pre-treatment steps.

Groups	Analytes	Abbreviation	Analytes	Abbreviation
A group	aflatoxin B_1_	AFB_1_	dihydrolysergol	DH-LYS
	aflatoxin B_2_	AFB_2_	elymoclavine	Ecl
	aflatoxin G_1_	AFG_1_	Ergine	Ergine
	aflatoxin G_2_	AFG_2_	ergocornine	Eco
	aflatoxin M_1_	AFM_1_	ergocorninine	Econ
	aflatoxin M_2_	AFM_2_	ergocristine	Ecr
	ochratoxin A	OTA	ergocristinine	Ecrn
	ochratoxin B	OTB	dihydroergocristine	DH-Ecr
	fumonisin B_1_	FB_1_	ergokryptine	Ek
	fumonisin B_2_	FB_2_	ergokryptinine	Ekn
	fumonisin B_3_	FB_3_	ergometrine	Em
	T-2 toxin	T2	ergometrinine	Emn
	HT-2 toxin	HT2	ergosinine	Esn
	T-2 triol toxin	T2(OH)3	ergotamine	Et
	beauvericin	BEA	ergotaminine	Etn
	enniatin A	EnA	gliotoxin	GLIO
	enniatin A_1_	EnA_1_	mycophenolic acid	MPA
	enniatin B	EnB	penicillic acid	PCA
	enniatin B_1_	EnB_1_	roquefortine C	RC
	neosolaniol	NEO	sterigmatocystin	STG
	15-acetoxyscirpenol	15AS	cyclopiazonic acid	CPA
	4,15-diacetoxyscirpenol	DAS	citrinin	CIT
	agroclavine	Acl		
B group	zearalanone	ZAN	altenuene	ALT
	zearalenone	ZEN	tenuazonic acid	TeA
	alpha-zearalenol	α-ZEL	altertoxin I	AXT I
	beta-zearalenol	β-ZEL	tentoxin	TEN
	alpha-zearalanol	α-ZAL	moniliformin	MON
	beta-zearalanol	β-ZAL	patulin	PAT
	alternariol	AOH	ochratoxin- alpha	OTα
	alternariol monomethyl ether	AME		
C group	deoxynivalenol	DON	nivalenol	NIV
	deoxynivalenol 3-glucuronide	D3G	3-acetyldeoxynivalenol	3AcDON
	deepoxy-deoxynivalenol	DOM	15-acetyldeoxynivalenol	15AcDON
	fusarenon-X	FusX		
IS	^13^C-aflatoxin B_1_	^13^C-AFB_1_	^13^C-sterigmatocystin	^13^C-STG
	^13^C-aflatoxin B_2_	^13^C-AFB_2_	^13^C-citrinin	^13^C-CIT
	^13^C-aflatoxin G_1_	^13^C-AFG_1_	^13^C-zearalanone	^13^C-ZEN
	^13^C-aflatoxin G_2_	^13^C-AFG_2_	^13^C-patulin	^13^C-PAT
	^13^C-aflatoxin M_1_	^13^C-AFM_1_	^13^C-alternariol	^13^C-AOH
	^13^C-ochratoxin A	^13^C-OTA	^13^C-alternariol monomethyl ether	^13^C-AME
	^13^C-T-2 toxin	^13^C-T2	^13^C-tenuazonic acid	^13^C-TeA
	^13^C-HT-2 toxin	^13^C-HT2	tentoxin-d_3_	TEN-d_3_
	^13^C-fumonisin B_1_	^13^C-FB_1_	^13^C-deoxynivalenol	^13^C-DON
	^13^C-fumonisin B_2_	^13^C-FB_2_	^13^C-deoxynivalenol	^13^C-D3G
	^13^C-fumonisin B_3_	^13^C-FB_3_	^13^C-nivalenol	^13^C-NIV
	^13^C-4,15-diacetoxyscirpenol	^13^C-DAS	^13^C-3-acetyldeoxynivalenol	^13^C-3AcDON
	^13^C-mycophenolic acid	^13^C-MPA	^13^C-15-acetyldeoxynivalenol	^13^C-15AcDON
	^13^C-roquefortine C	^13^C-RC		

## Data Availability

All data used in this work is available either within the article or in the supporting information file.

## Supplementary Materials

The following supporting information can be downloaded at: https://www.mdpi.com/article/10.3390/toxics12060395/s1, Figure S1: RA and ME assessment of mycotoxins pre-purification and post-purification by SPE (HLB or EMR). Part (B1) and (B2) are the 15 analytes from group B, and Part (C) is the 7 analytes from group C; Figure S2: LC-MS/MS extracted ion chromatograms of bank plasma samples and plasma matrices at their respective LOQ; Figure S3: Short term stability study at 4 °C and 25 °C for 3 h, 6 h, 1 day, 3 days and 5 days storage period (%); Figure S4: Representative S-MRM chromatograms of positive plasma samples; Table S1: A summary of mycotoxins HBM in human plasma, serum, and blood using LC-MS/MS; Table S2: Chemical properties of 67 mycotoxins analyzed in this study; Table S3: MS parameters of 67 mycotoxins; Table S4: Evaluation of the developed method for sensitivity, ME, RM, linearity, and precision of 67 mycotoxins; Table S5: Short term stability study at 4 °C and 25 °C for 3 h, 6 h, 1 day, 3 days, and 5 days storage period (%); Table S6: Comparison between previously reported LODs and LODs determined in this study for mycotoxins; Table S7: Mycotoxins levels in individual plasma samples using the UHPLC-MS/MS method. The references [62,63,64,65,66,67] are cited in the Supplementary materials.
