# Supplementary material for: High-Coverage UHPLC-MS/MS Analysis of 67 Mycotoxins in Plasma for Male Infertility Exposure Studies
Source: Toxics. 2024 May 28;12(6):395. doi: 10.3390/toxics12060395 (PMC11209182; doi:10.3390/toxics12060395)
Supplement: Supplementary file 1 [file toxics-12-00395-s001.zip › toxics-2972439-supplementary.pdf]

High-Coverage UHPLC-MS/MS Analysis of 67 Mycotoxins in Plasma for Male Infertility Exposure Studies

Xiao Ning[a, b], *Lulu Wang*[b], *Jia-Sheng Wang*[c], *Jian Ji*[a], *Shaoming Jin*[b], *Jiadi Sun*[a], *Yongli Ye*[a], *Shenghui Mei*[d,e], *Yinzhi Zhang*[a], *Jin Cao*[b,*], *and Xiulan Sun*[a,*]

[a] School of Food Science and Technology, International Joint Laboratory on Food Safety, Synergetic Innovation Center of Food Safety and Quality Control, Jiangnan University, Wuxi, Jiangsu 214122, P.R. China

[b] Key Laboratory of Food Quality and Safety for State Market Regulation, National Institute of Food and Drug Control, Beijing 100050, China

[c] Department of Environmental Health Science, College of Public Health, University of Georgia, Athens, GA, 30602, USA

[d] Department of Pharmacy, Beijing Tiantan Hospital, Capital Medical University, Beijing 100070, China

[e] Department of Clinical Pharmacology, School of Pharmaceutical Sciences, Capital Medical University, Beijing 100069, China

*Correspondence authors

sxlzzz@jiangnan.edu.cn (Xiulan Sun), caojin@nifdc.org.cn (Jin Cao)

Tel.: +86-0510-8591-2330 (Xiulan Sun.), +86-010-6709-5070 (Jin Cao)

1. Materials and Methods

1.1 Samples

A total of 184 men were investigated between December 2014 and August 2015. Inclusion criteria of case group (*n*=89) were couples who cohabit after marriage for more than one year without the use of contraception failed to conceive or have children and aged between 21 and 49 years. Exclusion criteria were as follows: abnormal secondary sex characteristics, sexual dysfunction, and genitourinary infection. The control group (*n*=95) was selected who went to the hospital for normal physical examination, had normal secondary sex characteristics and sexual function and did not have infertility. The study was approved by the ethics committee of the Beijing Obstetrics and Gynecology Hospital, Capital Medical University (ethics number 20141201). All participants had signed written informed consent.

1.2 Mobile phase gradients and ion source parameters

Group A. 45 mycotoxins were separated on a Poroshell 120 EC-C18 column (2.1×150mm, 2.7μm, Agilent). The eluent was composed of 0.1% FA with 1 mM NH_4HCO_2(A) and ACN (B). The gradient elution was performed as follows: 10% B (initial), 10%–30% B (1–5 min), 30%–95% B (5–11 min), 95% B (11–15 min), 95%–10% B (15–15.1 min), and 10% B (15.1–18 min). The flow rate was 0.3 mL/min. The column temperature was kept at 40°C and 10 μL of each sample was injected for analysis. Positive electrospray ionization was set at 450°C with an ion spray voltage of 2000 V. Curtain gas, nebulizer gas, aux gas, and collision gas were set at 40, 40, 80, and 10 psi, respectively.

Group B. The eluent is composed of 0.1 mM CH_3COONH_4 (A) and ACN (B), and the chromatographic separation conditions of 15 mycotoxins in group B are basically the same as those in group A. Negative electrospray ionization was set at 500°C with an ion spray voltage of -1800 V. Curtain gas, nebulizer gas, aux gas, and collision gas were set at 40, 40, 80, and 10 psi, respectively.

Group C. Chromatographic separation of the group DON and its derivatives (DON, 3AcDON,

15AcDON, D3G, DOM, FusX, and NIV) was performed on a Poroshell 120 EC-C18 column (2.1×150 mm, 2.7 μm, Agilent) with a mobile phase consisting of 0.01% HAc (A) and ACN (B) at a flow rate of 0.25 mL/min. The following elution gradient was applied: 2% B (initial), 2%–25% B (0–5 min), 25%–30% B (5–8 min), 30%–95% B (8–9 min), 95% B (9–11 min), 95%–2% B (11–11.1 min), and 2% B (11.1–14 min). The column temperature was kept at 40 °C, and the injection volume was 10 μL. Negative electrospray ionization was set at 350°Cwith an ion spray voltage of -2400 V. Curtain gas, nebulizer gas, aux gas, and collision gas were set at 40, 40, 50, and 9 psi, respectively.

1.3 Calculations of apparent recoveries (R_A) and matrix effects (M_E) of mycotoxins during the sample preparation process.

$$\mathrm{M_E}\ (\%) = \frac{B}{A} \times 100 \quad (1)$$

$$\mathrm{R_A}(\%) = \frac{C}{A} \times 100 \quad (2)$$

where A represents the slope of a calibration curve prepared in neat solvent; B represents the slope of a matrix-matched calibration curve prepared by spiking blank samples after sample preparation; and C represents the slope of a matrix-matched calibration curve prepared by spiking blank samples before sample preparation.

1.4 Optimization of other chromatographic conditions

DON and its derivatives exhibit similar structures and properties. Isocratic elution conditions did not achieve adequate separation; the positional isomers 3AcDON and 15AcDON exhibited a common precursor ion (m/z 397.3) and similar product ions. Thus, to avoid peak overlapping and to achieve accurate quantification, a gradient elution method was applied for these analytes. Accordingly, a flow rate of 0.25 mL/min and a mild gradient elution of 25–30% B from 5 to 8 min afforded the optimal separation of 3AcDON and 15AcDON.

A scheduled multiple reaction monitoring (SMRM) acquisition method was developed after the

determination of the elution times of each mycotoxin. In this mode, transitions between a precursor ion and the two most abundant fragment ions were chosen for each analyte. A maximized dwell time, an optimal cycle time, and the highest possible duty cycle for each MRM ensured that the analytical precision was maintained at higher multiplexing.

1.5 MS/MS condition optimization

To further improve performance, the key parameters that affect the ionization procedure such as ionization mode and the curtain gas were manually optimized in a stepwise manner. The ion spray voltage (ISV) was tested over a range of 1400–2600 V, the source temperature was tested over a range of 350–650 °C, and the pressures of the nebulizer gas (gas1) and auxiliary gas (gas2) were tested at 30–80 psi. The peak areas of the 67 mycotoxins were affected differently by these ion source parameters.

As shown in Figure 2A, as the ISV increased, the peak areas of FBs, T2, HT2, T2(OH)3, cyclohexaester peptides (BEA and enniatins), and diacetoxyscirpenols (DAS, NEO, and 15AS) increased substantially, whereas the peak areas of AFs, STG, CPA, MPA, CIT, PCA, and RC decreased slightly. For OTs, ergot alkaloids, and their respective inin-epimers, the optimal ISV was achieved at 2000 V, and the peak areas decreased at both higher and lower ISV values. Therefore, 2000 V was chosen as the ISV for analysis of group A mycotoxins.

Although the signal intensity of most analytes increased with increasing temperature, the signal intensities of T2, HT2, T2(OH)3, cyclohexaester peptides, diacetoxyscirpenols, and GLIO decreased greatly when the temperature was higher than 450°C. Therefore, 450°C was selected as the optimum temperature.

The peak areas of T2, HT2, T2(OH)3, diacetoxyscirpenols, and GLIO were directly proportional to the pressure of gas1, whereas other analytes showed optimal sensitivity when the pressure of gas1 reached 40 psi. Upon increase in gas2 pressure, the peak areas of most analytes increased, whereas the sensitivity of detection of cyclohexaester peptides and diacetoxyscirpenols decreased. To accommodate the requirements

of most mycotoxins, the pressure of gas1 was maintained at 40 psi and that of gas2 was maintained at 80 psi; the sensitivity of detection of all analytes was adequate under these conditions.

Regarding group B mycotoxins, as the ISV value increased, the peak area of all analytes uniformly increased at first and then decreased. Optimal performance was obtained using an ISV of −1800 V at 500 °C, and the pressures of gas1 and gas2 were set at 40 and 80 psi, respectively. For compounds in group C, the relationships between the ion source parameters and the response values of the analytes were similar to those in group B. The peak areas of most compounds in group C increased slightly upon increases in gas2 pressure; in contrast, the area of the peak representing 15AcDON significantly decreased. Accordingly, a moderate value of 50 psi was used for the pressure of gas2. Detailed results are shown in Figures 2B and 2C.

Captions

Table S1 A summary of mycotoxins HBM in human plasma, serum, and blood using LC-MS/MS.

Table S2 Chemical properties of 67 mycotoxins analyzed in this study.

Table S3 MS parameters of 67 mycotoxins.

Table S4 Evaluation of the developed method for sensitivity, M_E, R_M, linearity, and precision of 67 mycotoxins.

Table S5 Short term stability study at 4ºC and 25ºC for 3 h, 6 h ,1 day, 3 days and 5 days storage period (%).

Table S6 Comparison between previously reported LODs and LODs determined in this study for mycotoxins.

Table S7 Mycotoxins levels in individual plasma samples using the UHPLC-MS/MS method.

Figure S1 R_A and M_E assessment of mycotoxins pre-purification and post-purification by SPE (HLB or EMR). Part (B1) and (B2) are the 15 analytes from group B, and Part (C) is the 7 analytes from group C.

Figure S2 LC-MS/MS extracted ion chromatograms of bank plasma samples and plasma matrices at their respective LOQ.

Figure S3 Short term stability study at 4ºC and 25ºC for 3 h, 6 h ,1day, 3 days and 5 days storage period (%).

Figure S4 Representative S-MRM chromatograms of positive plasma samples.

Table S1

Country/ Samples	Analyte	Detection (%)	LOD (μg/L)	LOQ (μg/L)	Preparation method	Mean and/or [Range] (μg/L)	Year/Ref
Spain	DOM	0	1.35		400 μL plasma;	ND	[37]
n=79(40/7/2/30)	AFG2	0	0.35		Dilute, clean-up by SPE, evaporate and reconstitution	ND	
C[1]/ADHD/ASD/DD	AFM1	0	0.18			ND	
Children	AFG1	0	0.07			ND	
	AFB2	0	0.07			ND	
	AFB1	0	0.04			ND	
	HT2	0	2.70			ND	
	OTB	11.4/0/0/0	0.4	1		C: 0.57 [0.4-0.8]	
	T2	0	0.20			ND	
	ZAN	0	1.80			ND	
	OTA	92.5/0/0/76.7	0.40	2		C: 2.42 [3.29 ± 2.70]<br>DD:1.04 [1.90 ± 3.48]	
	STG	97.5/0/0/100	0.20	1		C: 1.41[1.41 ± 0.38]<br>DD:1.54 [1.53 ± 0.33]	
	NIV	0	9.10			ND	
	DON	0	1.94			ND	
	FusX	0	1.95			ND	
	NEO	0	0.18			ND	
	3-AcDON	0	0.70			ND	
	15-AcDON	0	1.20			ND	
	DAS	0	0.15			ND	
Tunisia	PAT	20/30	1.1	2.3	1 mL plasma;	11.62	[56]
n=100 (50/50)C[1]/CRCA	CIT	34/38	0.04	0.09	Extraction and QuEChERS, evaporation and reconstitution	0.49	
Italy	ZEN	-	2.5	5	A portion of serum;	＜LOQ	[62]
n=110(52/31/27)ASD/CS/C[2]	α-ZEL	-	2.5	5	Extraction by LLE, evaporation and reconstitution	＜LOQ	
	β-ZEL	-	2.5	5		＜LOQ	
China	OTA	27.7	0.04	0.1	200 μL plasma;	1.21[0.312–9.18]	[51]
n=260	FB1	2.7	0.2	0.5	Extraction, dried by nitrogen and reconstitution	0.69 [0.305–0.993]	
(Rural residents age 18-66 years)	DON	2.3	0.5	1		2.60 [1.39–5.53]	
	ZEN	6.5	0.05	0.1		0.16 [0.063–0.418]	
	ZAN	1.2	0.03	0.1		0.26 [0.164–0.346]	

Country/ Samples	Analyte	Detection (%)	LOD (μg/L)	LOQ (μg/L)	Preparation method	Mean and/or [Range] (μg/L)	Year/Ref
Bangladesh n=104(Young adults)	CIT	90	0.07	0.15	1 mL plasma; Extraction, dried by nitrogen and reconstitution	0.22 [n.d.–2.70]	[55]
	DH-CIT	85	0.15	0.3		0.31 [n.d.–1.44]	
	OTA	100	0.05	0.10		0.72 [0.10–6.63]	
	OTα	98	0.05	0.10		0.38 [n.d.–0.99]	
Czech Republic n=50 (Renal tumours patients)	CIT	100	0.07	0.15	1 mL plasma； Extraction, dried by nitrogen and reconstitution	0.061 [0.02–0.18]	[63]
	DH-CIT	0	0.15	0.3		ND	
	OTA	48	0.04	0.1		0.15 [0.04–830]	
USA n=48 overweight/obese women (52 ± 9 years)	ZEN	85.4	0.07		500μL serum; Extraction and clean-up by SPE, evaporation and reconstitution	0.087	[64]
	α-ZEL	6.3	0.07			ND	
	β-ZEL	35.4	0.07			0.089	
	α-ZAL	16.7	0.07			ND	
	ZAN	31.3	0.07			0.102	
	β-ZAL	8.3	0.07			ND	
China n=60 (30/30)C[1]/HCC	AFB1	13/33	0.07	0.25	200 μL plasma; Enzymatic treatment, extraction, evaporation and reconstitution	[0.95–1.78]/[1.23–4.56]	[65]
	AFB2	17/23	0.05	0.21		[1.37–3.89]/[1.16–3.75]	
	AFG1	3/3	0.13	0.43		0.61/0.55	
	AFG2	3/3	0.15	0.38		0.43/0.46	
	AFM1	3/0	0.16	0.41		0.57/ND	
	STG	13/40	0.05	0.22		[0.88–2.05]/[1.06–3.23]	
	PAT	0/0	0.35	0.83		ND/ND	
	CIT	0/3	0.18	0.44		ND/0.63	
	FB1	3/7	0.41	0.92		1.92/[1.35–2.78]	
	FB2	3/3	0.39	0.87		2.03/1.57	
	OTA	0/3	0.15	0.46		ND/0.83	
	BEA	0/0	0.02	0.04		ND/ND	
Italy n=213 (Children with Autism)	AFB1	22.9	0.005	0.01	1mL serum; Enzymatic treatment, extraction, QuEChERS and then dried by nitrogen and reconstitution	0.01 [0–0.73]	[66]
	AFM1	50.2	0.11	0.22		0.11 [0–1.91]	
	DON	19.5	2.5	5		1.0 [0–27.9]	
	DOM	13.1	2.5	5		0.3 [0–12.7]	
	FB1	13.7	1.5	3		0.7 [0–5.6]	
	GLIO	21.2	5.5	11		2.3 [0–28.4]	
	OTA	82.9	0.08	0.16		0.36 [0–1.76]	
	ZEN	5.4	0.5	1		0.1 [0–3.9]	
Germany	OTA	100	0.012	0.05	100 μL whole blood or serum;	0.204	[52]

Country/ Samples	Analyte	Detection (%)	LOD (μg/L)	LOQ (μg/L)	Preparation method	Mean and/or [Range] (μg/L)	Year/Ref
n=50(Healthy volunteers)	EnB	100	0.0012	0.01	The DBS and DSS were extracted, then dryness under reduced pressure and reconstitution	0.036[0.014–0.11]	
	AFB1	0	0.0012	0.05		ND	
	AFB2	0	0.0013	0.05		ND	
	AFG1	0	0.0021	0.1		ND	
	AFG2	0	0.0037	0.125		ND	
	AFM1	0	0.0017	0.1		ND	
	ALT	0	0.147	0.5		ND	
	AME	0	0.146	0.5		ND	
	AOH	0	0.142	0.5		ND	
	BEA	0	0.014	0.05		ND	
	CIT	0	0.066	0.25		ND	
	DH-CIT	0	0.268	1.0		ND	
	DON	0	0.263	1.0		ND	
	D3G	0	1.287	5.0		ND	
	EnA	0	0.0016	0.01		ND	
	EnA_1	0	0.0055	0.025		ND	
	EnB_1	0	0.0044	0.025		ND	
	FB_1	0	0.521	2.5		ND	
	HT2	0	1.344	5.0		ND	
	OTα	0	0.014	0.05		ND	
	T2	0	0.227	0.1		ND	
	ZAN	0	0.273	1.0		ND	
	ZEN	0	0.294	1.0		ND	
Germany n=50 (Participants age 18-60 years)	OTA	100	0.006	0.021	100 μL whole blood; DBS was extracted, then dryness under reduced pressure and reconstitution	0.211 [0.071–0.383]	[67]

Note: ND means not detected.C^1: control group; ADHD: attention deficit hyperactivity disorder, ASD:autism spectrum disorder; DD:digestive disorders; CRCA:colorectal cancer; HCC:hepatocellular carcinoma; ASD:autism spectrum disorder, CS:Control (siblings),C^2:Control (pure).

Table S2

Groups	Analytes	CAS	Molecular formula	Structure
A	aflatoxin B_1	1162-65-8	$C_{17}H_{12}O_6$	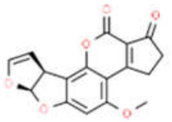
	aflatoxin B_2	7220-81-7	$C_{17}H_{14}O_6$	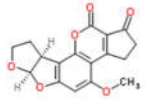
	aflatoxin G_1	1165-39-5	$C_{17}H_{12}O_7$	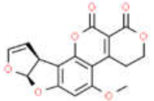
	aflatoxin G_2	7241-98-7	$C_{17}H_{14}O_7$	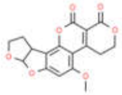
	aflatoxin M_1	6795-23-9	$C_{17}H_{12}O_7$	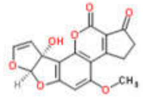
	aflatoxin M_2	6885-57-0	$C_{17}H_{14}O_7$	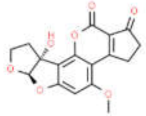
	ochratoxin A	303-47-9	$C_{20}H_{18}ClNO_6$	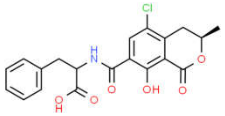

Groups	Analytes	CAS	Molecular formula	Structure
	ochratoxin B	4825-86-9	$C_{20}H_{19}NO_6$	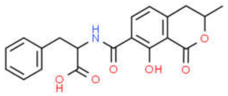
	fumonisin B_1	116355-83-0	$C_{34}H_{59}NO_{15}$	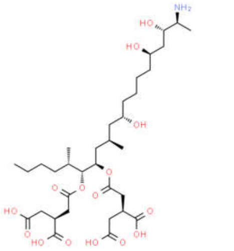
	fumonisin B_2	116355-84-1	$C_{34}H_{59}NO_{14}$	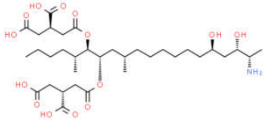
	fumonisin B_3	1422359-85-0	$C_{34}H_{59}NO_{14}$	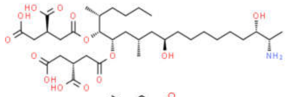
	T-2 toxin	21259-20-1	$C_{24}H_{34}O_9$	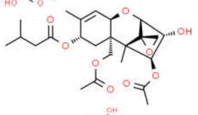
	HT-2 toxin	26934-87-2	$C_{22}H_{32}O_8$	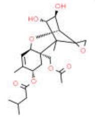
	T-2 triol toxin	34114-98-2	$C_{20}H_{30}O_7$	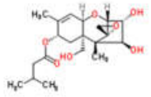
	beauvericin	26048-05-5	$C_{45}H_{57}N_3O_9$	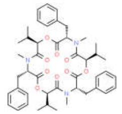

Groups	Analytes	CAS	Molecular formula	Structure
	enniatin A	2503-13-1	$C_{36}H_{63}N_3O_9$	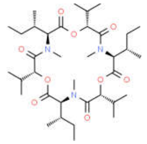
	enniatin A_1	4530-21-6	$C_{35}H_{61}N_3O_9$	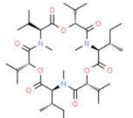
	enniatin B	917-13-5	$C_{33}H_{57}N_3O_9$	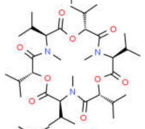
	enniatin B_1	19914-20-6	$C_{34}H_{59}N_3O_9$	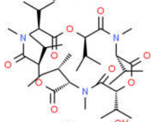
	neosolaniol	36519-25-2	$C_{19}H_{26}O_8$	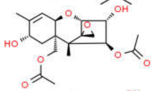
	15-acetoxyscirpenol	2623-22-5	$C_{17}H_{24}O_6$	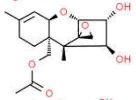
	4,15-diacetoxyscirpenol	2270-40-8	$C_{19}H_{26}O_7$	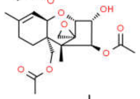
	agroclavine	548-42-5	$C_{16}H_{18}N_2$	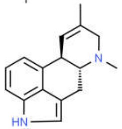
	dihydrolysergol	18051-16-6	$C_{16}H_{20}N_2O$	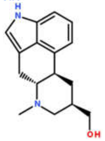

Groups	Analytes	CAS	Molecular formula	Structure
	elymoclavine	548-43-6	$C_{16}H_{18}N_2O$	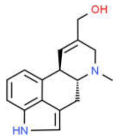
	Ergine	478-94-4	$C_{16}H_{17}N_3O$	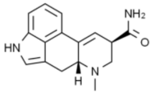
	ergocornine	564-36-3	$C_{31}H_{39}N_5O_5$	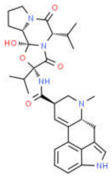
	ergocorninine	564-37-4	$C_{31}H_{39}N_5O_5$	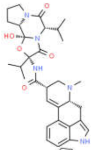
	ergocristine	511-08-0	$C_{35}H_{39}N_5O_5$	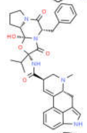
	ergocristinine	511-07-9	$C_{35}H_{39}N_5O_5$	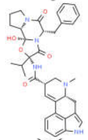
	dihydroergocristine	17479-19-5	$C_{35}H_{41}N_5O_5$	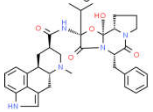
	ergokryptine	511-09-1	$C_{32}H_{41}N_5O_5$	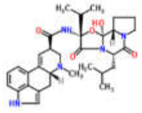

Groups	Analytes	CAS	Molecular formula	Structure
	ergokryptinine	511-10-4	$C_{32}H_{41}N_5O_5$	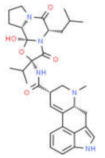
	ergometrine	60-79-7	$C_{19}H_{23}N_3O_2$	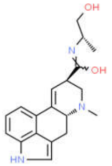
	ergometrinine	479-00-5	$C_{19}H_{23}N_3O_2$	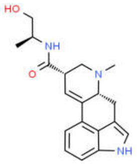
	ergosinine	561-94-4	$C_{30}H_{37}N_5O_5$	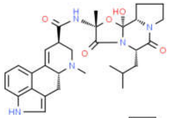
	ergotamine	113-15-5	$C_{33}H_{35}N_5O_5$	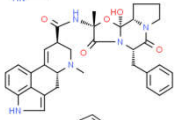
	ergotaminine	639-81-6	$C_{30}H_{35}N_5O_5$	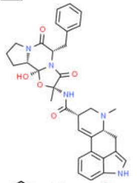
	gliotoxin	67-99-2	$C_{13}H_{14}N_2O_4S_2$	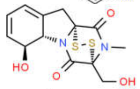
	mycophenolic acid	24280-93-1	$C_{17}H_{20}O_6$	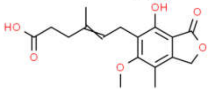

Groups	Analytes	CAS	Molecular formula	Structure
	penicillic acid	90-65-3	$C_8H_{10}O_4$	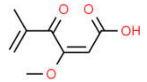
	roquefortine C	58735-64-1	$C_{22}H_{23}N_5O_2$	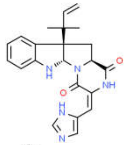
	sterigmatocystin	10048-13-2	$C_{18}H_{12}O_6$	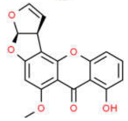
	cyclopiazonic acid	18172-33-3	$C_{20}H_{20}N_2O_3$	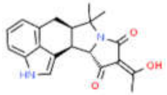
	citrinin	518-75-2	$C_{13}H_{14}O_5$	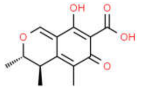
B	zearalanone	5975-78-0	$C_{18}H_{24}O_5$	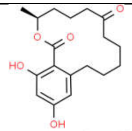
	zearalenone	17924-92-4	$C_{18}H_{22}O_5$	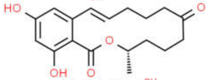
	alpha-zearalenol	36455-72-8	$C_{18}H_{24}O_5$	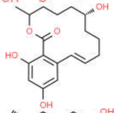
	beta-zearalenol	71030-11-0	$C_{18}H_{24}O_5$	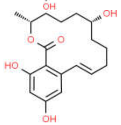

Groups	Analytes	CAS	Molecular formula	Structure
	alpha-zearalanol	26538-44-3	$C_{18}H_{26}O_5$	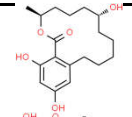
	beta-zearalanol	42422-68-4	$C_{18}H_{26}O_5$	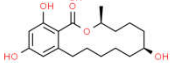
	alternariol	641-38-3	$C_{14}H_{10}O_5$	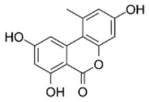
	alternariol monomethyl ether	23452-05-3	$C_{15}H_{12}O_5$	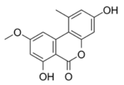
	altenuene	29752-43-0	$C_{15}H_{16}O_6$	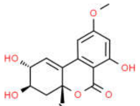
	tenuazonic acid	610-88-8	$C_{10}H_{15}NO_3$	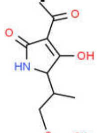
	altertoxin I	56258-32-3	$C_{20}H_{16}O_6$	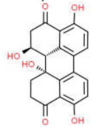
	tentoxin	28540-82-1	$C_{22}H_{30}N_4O_4$	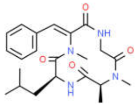
	moniliformin	71376-34-6	C_4HNaO_3	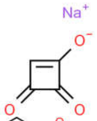
	patulin	149-29-1	$C_7H_6O_4$	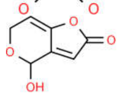

Groups	Analytes	CAS	Molecular formula	Structure
	ochratoxin- alpha	19165-63-0	$C_{11}H_{9}ClO_{5}$	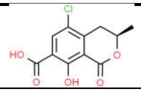
	deoxynivalenol	51481-10-8	$C_{15}H_{20}O_{6}$	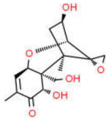
	Deoxynivalenol 3-glucuronide	1000000-13-4	$C_{21}H_{28}O_{12}$	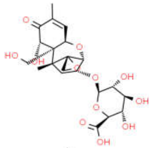
	deepoxy-deoxynivalenol	88054-24-4	$C_{15}H_{20}O_{5}$	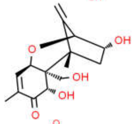
C	fusarenon-X	23255-69-8	$C_{17}H_{22}O_{8}$	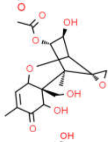
	nivalenol	23282-20-4	$C_{15}H_{20}O_{7}$	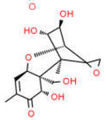
	3-acetyldeoxynivalenol	50722-38-8	$C_{17}H_{22}O_{7}$	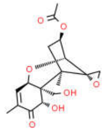
	15-acetyldeoxynivalenol	88337-96-6	$C_{17}H_{22}O_{7}$	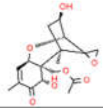
IS	^{13}C-aflatoxin B_1	1217449-45-0	$^{13}C_{17}H_{12}O_{6}$	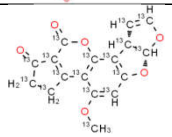

Groups	Analytes	CAS	Molecular formula	Structure
	^{13}C-aflatoxin B_2	1217470-98-8	$^{13}C_{17}H_{14}O_6$	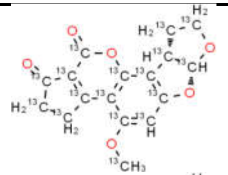
	^{13}C-aflatoxin G_1	1217444-07-9	$^{13}C_{17}H_{12}O_7$	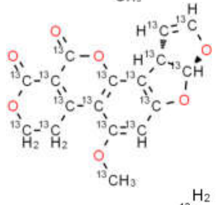
	^{13}C-aflatoxin G_2	1217462-49-1	$^{13}C_{17}H_{14}O_7$	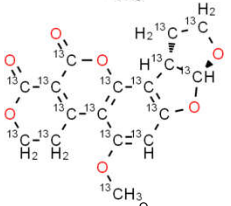
	^{13}C-aflatoxin M_1	2707441-97-0	$^{13}C_{17}H_{12}O_7$	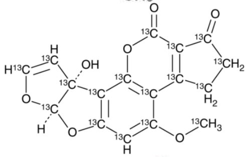
	^{13}C-ochratoxin A	911392-42-2	$^{13}C_{20}H_{18}ClNO_6$	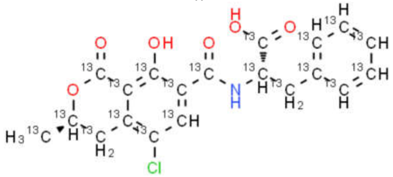
	^{13}C-T-2 toxin	21259-20-1(unlabeled)	$^{13}C_{24}H_{34}O_9$	/
	^{13}C-HT-2 toxin	1486469-92-4	$^{13}C_{22}H_{32}O_8$	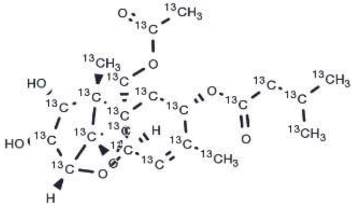

Groups	Analytes	CAS	Molecular formula	Structure
	^{13}C-fumonisin B_1	1217458-62-2	$^{13}C_{34}H_{59}NO_{15}$	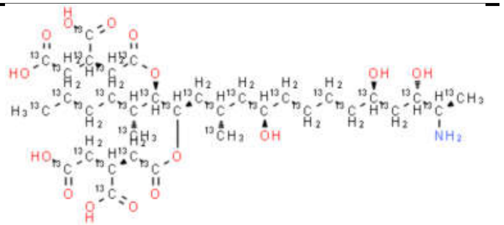
	^{13}C-fumonisin B_2	1217481-36-1	$^{13}C_{34}H_{59}NO_{14}$	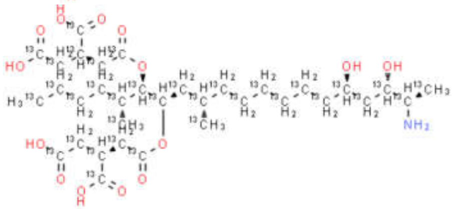
	^{13}C-fumonisin B_3	1217494-88-6	$^{13}C_{34}H_{59}NO_{14}$	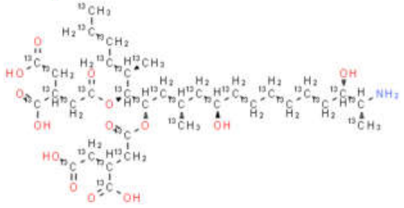
	^{13}C-4,15-diacetoxyscirpenol	2270-40-8(unlabeled)	$^{13}C_{19}H_{26}O_7$	/
	^{13}C-mycophenolic acid	1202866-92-9	$^{13}C_{17}H_{20}O_6$	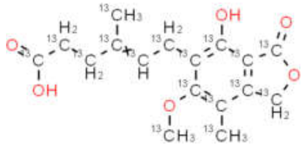
	^{13}C-roquefortine C	58735-64-1 (unlabeled)	$^{13}C_{22}H_{23}N_5O_2$	/
	^{13}C-sterigmatocystin	10048-13-2(unlabeled)	$^{13}C_{18}H_{12}O_6$	/
	^{13}C-citrinin	1329611-85-9	$^{13}C_{13}H_{14}O_5$	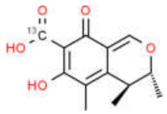

Groups	Analytes	CAS	Molecular formula	Structure
	^{13}C-zearalanone	911392-43-3	$^{13}C_{18}H_{22}O_5$	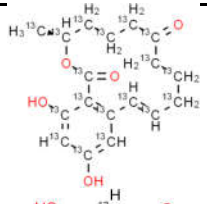
	^{13}C-patulin	1353867-99-8	$^{13}C_7H_6O_4$	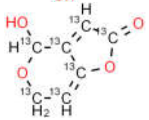
	^{13}C-alternariol	26894-49-5(unlabeled)	$^{13}C_{14}H_{10}O_5$	/
	^{13}C-alternariol monomethyl ether	23452-05-3(unlabeled)	$^{13}C_{14}H_{10}O_5$	/
	^{13}C-tenuazonic acid	1486471-66-2	$^{13}C_{10}H_{15}NO_3$	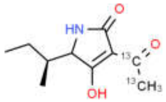
	tentoxin-d$_3$	28540-82-1(unlabeled)	$C_{22}H_{27}D_3N_4O_4$	/
	^{13}C-deoxynivalenol	911392-36-4	$^{13}C_{15}H_{20}O_6$	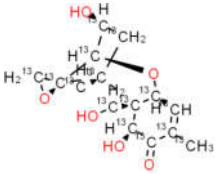
	^{13}C-deoxynivalenol 3-glucuronide	1000000-13-4(unlabeled)	$^{13}C_{21}H_{28}O_{12}$	/
	^{13}C-nivalenol	911392-40-0	$^{13}C_{15}H_{20}O_7$	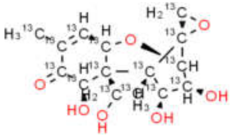
	^{13}C-3-acetyldeoxynivalenol	1217476-81-7	$^{13}C_{17}H_{22}O_7$	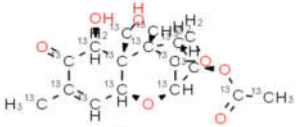
	^{13}C-15-acetyldeoxynivalenol	911392-39-7	$^{13}C_{17}H_{22}O_7$	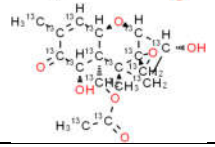

Table S3

Groups	Analytes	Molecular ion	Retention time (min)	m/z	Quantifier	Collision energy (V)	Qualifier	Collision energy (V)	IS
	AFB_1	$[M+H]^+$	7.85	313.1	285	33	241	51	$^{13}C\text{-}AFB_1$
	AFB_2	$[M+H]^+$	7.52	315.1	287	36	259.1	41	$^{13}C\text{-}AFB_2$
	AFG_1	$[M+H]^+$	7.48	329	243	37	200.1	55	$^{13}C\text{-}AFG_1$
	AFG_2	$[M+H]^+$	7.08	331.1	313	33	245	42	$^{13}C\text{-}AFG_2$
	AFM_1	$[M+H]^+$	6.56	329	273	31	259	33	$^{13}C\text{-}AFM_1$
	AFM_2	$[M+H]^+$	7.04	331.1	257	40	245	45	$^{13}C\text{-}AFM_1$
	OTA	$[M+H]^+$	9.47	404	239	37	358	20	$^{13}C\text{-}OTA$
	OTB	$[M+H]^+$	8.75	370.1	205	26	324	19	$^{13}C\text{-}OTA$
	FB_1	$[M+H]^+$	7.57	722.4	704.2	41	334.3	48	$^{13}C\text{-}FB_1$
	FB_2	$[M+H]^+$	8.26	706.4	354.3	43	336.4	50	$^{13}C\text{-}FB_2$
	FB_3	$[M+H]^+$	7.95	706.4	318.3	51	354.3	43	$^{13}C\text{-}FB_3$
	T2	$[M+NH_4]^+$	9.22	484.3	215	22	305.2	18	$^{13}C\text{-}T2$
	HT2	$[M+NH_4]^+$	8.23	442.3	263.2	16	215.2	16	$^{13}C\text{-}HT2$
	T2（OH）3	$[M+NH_4]^+$	4.59	400.1	215.2	20	105.1	57	$^{13}C\text{-}T2$
A	BEA	$[M+NH_4]^+$	12.16	801.5	784.4	24	134.1	95	$^{13}C\text{-}DAS$
	EnA	$[M+NH_4]^+$	12.77	699.5	210.3	42	555.4	38	$^{13}C\text{-}DAS$
	EnA_1	$[M+NH_4]^+$	12.60	685.5	668.2	24	210.2	39	$^{13}C\text{-}AFB_2$
	EnB	$[M+NH_4]^+$	11.94	657.5	196.2	40	640.2	23	$^{13}C\text{-}AFB_2$
	EnB_1	$[M+NH_4]^+$	12.22	671.5	196.1	39	654.3	23	$^{13}C\text{-}AFB_2$
	NEO	$[M+NH_4]^+$	4.59	400.2	305.1	15	159	24	$^{13}C\text{-}OTA$
	15AS	$[M+NH_4]^+$	6.19	342.2	107	21	265.2	13	$^{13}C\text{-}AFM_1$
	DAS	$[M+NH_4]^+$	7.77	384.1	104.9	48	307.1	15	$^{13}C\text{-}DAS$
	Acl	$[M+H]^+$	5.57	239.1	183.1	24	208.1	24	$^{13}C\text{-}OTA$
	DH-LYS	$[M+H]^+$	3.47	257.1	208.1	30	167	53	$^{13}C\text{-}OTA$
	Ecl	$[M+H]^+$	3.41	255.1	224.1	20	180.1	57	$^{13}C\text{-}AOH$
	Ergine	$[M+H]^+$	2.73	268.1	223.1	27	208.1	34	$^{13}C\text{-}RC$
	Eco	$[M+H]^+$	7.43	562.3	223.2	43	268.1	34	$^{13}C\text{-}AFB_2$
	Econ	$[M+H]^+$	7.65	562.3	223.2	39	268.1	34	$^{13}C\text{-}AFB_1$

Groups	Analytes	Molecular ion	Retention time (min)	m/z	Quantifier	Collision energy (V)	Qualifier	Collision energy (V)	IS
	Ecr	[M+H]$^{+}$	7.85	610.3	223.1	43	268.1	35	^{13}C-AFB$_2$
	Ecrn	[M+H]$^{+}$	8.12	610.2	268.1	34	223.1	41	^{13}C-AFB$_1$
	DH-Ecr	[M+H]$^{+}$	7.97	612.3	270.1	39	350.2	34	^{13}C-AFM$_1$
	Ek	[M+H]$^{+}$	7.72	576.3	223.1	44	267.9	33	^{13}C-AFB$_2$
	Ekn	[M+H]$^{+}$	7.95	576.3	223	42	267.9	35	^{13}C-AFB$_2$
	Em	[M+H]$^{+}$	3.25	326.1	208	38	223.1	32	^{13}C-AFM$_1$
	Emn	[M+H]$^{+}$	4.27	326.1	208	37	223.1	33	^{13}C-AFM$_1$
	Esn	[M+H]$^{+}$	7.13	548.3	223.1	46	268.1	46	^{13}C-OTA
	Et	[M+H]$^{+}$	7.36	582.3	223	39	268.2	32	^{13}C-AFB$_1$
	Etn	[M+H]$^{+}$	7.17	582.3	223.2	39	208	46	^{13}C-AFB$_1$
	GLIO	[M+H]$^{+}$	7.42	327	263.1	14	245.1	22	^{13}C-AFM$_1$
	MPA	[M+H]+	8.73	321.1	206.9	27	159	44	^{13}C-MPA
	PCA	[M+H]$^{+}$	4.95	171.1	125	18	97.1	22	^{13}C-RC
	RC	[M+H]$^{+}$	7.88	390.1	193.1	39	321.9	27	^{13}C-RC
	STG	[M+H]$^{+}$	9.84	325.1	280.9	49	310.1	34	^{13}C-STG
	CPA	[M+H]$^{+}$	10.24	337	196	25	182	26	^{13}C-STG
	CIT	[M+H]$^{+}$	8.50	251.1	233.1	17	205.1	27	^{13}C-CIT
B	ZAN	[M-H]$^{-}$	9.49	319.2	205.2	-29	275	-25	^{13}C-ZEN
	ZEN	[M-H]$^{-}$	9.54	317.2	174.9	-33	131	-38	^{13}C-ZEN
	α-ZEL	[M-H]$^{-}$	8.87	319.2	159.9	-40	274.9	-28	^{13}C-ZEN
	β-ZEL	[M-H]$^{-}$	8.44	319.2	159.9	-40	274.9	-28	^{13}C-ZEN
	α-ZAL	[M-H]$^{-}$	8.75	321.2	277.2	-30	303.2	-30	^{13}C-ZEN
	β-ZAL	[M-H]$^{-}$	8.34	321.2	277	-30	303	-30	^{13}C-ZEN
	AOH	[M-H]$^{-}$	8.05	257.1	215	-34	213	-31	^{13}C-AOH
	AME	[M-H]$^{-}$	9.50	271	256	-30	228	-39	^{13}C-AME
	ALT	[M-H]$^{-}$	7.21	291.1	214.1	-29	229.1	-20	TEN-d$_3$
	TeA	[M-H]$^{-}$	2.50	196.1	139.1	-27	112.1	-32	^{13}C-TeA
	AXT I	[M-H]$^{-}$	7.99	351.2	315.1	-22	333.1	-15	^{13}C-AME
	TEN	[M-H]$^{-}$	8.23	413.2	141.1	-25	271.1	-22	TEN-d$_3$
	MON	[M-Na]$^{-}$	1.07	97	41	-18	/	/	TEN-d$_3$
	PAT	[M-H]$^{-}$	2.35	153.1	80.9	-17	108.8	-13	^{13}C-PAT

Groups	Analytes	Molecular ion	Retention time (min)	m/z	Quantifier	Collision energy (V)	Qualifier	Collision energy (V)	IS
	OTα	$[M-H]^-$	5.07	255.1	211	-22	167	-34	^{13}C-OTA
C	DON	$[M+CH_3COO]^-$	5.05	355.1	295.1	-14	59.2	-50	^{13}C-DON
	D3G	$[M+CH_3COO]^-$	4.75	517.1	427.1	-30	457.1	-20	^{13}C-D3G
	DOM	$[M+CH_3COO]^-$	5.87	339.1	249.1	-16	59.1	-50	^{13}C-DON
	FusX	$[M+CH_3COO]^-$	6.15	413.3	262.9	-22	59.1	-50	^{13}C-DON
	NIV	$[M+CH_3COO]^-$	4.16	371.1	281.1	-30	59	-46	^{13}C-NIV
	3AcDON	$[M+CH_3COO]^-$	7.71	397.3	337.1	-13	307.2	-40	^{13}C-3AcDON
	15AcDON	$[M+CH_3COO]^-$	7.55	397.3	59	-40	337.1	-9	^{13}C-15AcDON
IS	^{13}C-AFB_1	$[M+H]^+$	/	330.1	255.1	53	/	/	/
	^{13}C-AFB_2	$[M+H]^+$	/	332.1	273.1	43	/	/	/
	^{13}C-AFG_1	$[M+H]^+$	/	346.1	212.1	59	/	/	/
	^{13}C-AFG_2	$[M+H]^+$	/	348	259.1	43	/	/	/
	^{13}C-AFM_1	$[M+H]^+$	/	346.1	288.1	35	/	/	/
	^{13}C-OTA	$[M+H]^+$	/	424.1	377.1	21	/	/	/
	^{13}C-T2	$[M+NH_4]^+$	/	508.2	198.1	31	/	/	/
	^{13}C-HT2	$[M+NH_4]^+$	/	464.3	113.1	65	/	/	/
	^{13}C-FB_1	$[M+H]^+$	/	756.4	356.4	59	/	/	/
	^{13}C-FB_2	$[M+H]^+$	/	740.5	340.3	55	/	/	/
	^{13}C-FB_3	$[M+H]^+$	/	740.5	340.3	53	/	/	/
	^{13}C-DAS	$[M+NH_4]^+$	/	403.2	244.1	23	/	/	/
	^{13}C-MPA	$[M+H]^+$	/	338.1	169.2	31	/	/	/
	^{13}C-RC	$[M+H]^+$	/	412.1	339.2	29	/	/	/
	^{13}C-STG	$[M+H]^+$	/	343	327	37	/	/	/
	^{13}C-CIT	$[M+H]^+$	/	264	202	36	/	/	/
	^{13}C-ZEN	$[M-H]^-$	/	335.1	139.9	-44	/	/	/
	^{13}C-PAT	$[M-H]^-$	/	159.9	86	-16	/	/	/
	^{13}C-AOH	$[M-H]^-$	/	271	168	-37	/	/	/
	^{13}C-AME	$[M-H]^-$	/	286	269	-41	/	/	/
	^{13}C-TeA	$[M-H]^-$	/	208.9	115	-35	/	/	/

Groups	Analytes	Molecular ion	Retention time (min)	m/z	Quantifier	Collision energy (V)	Qualifier	Collision energy (V)	IS
	TEN-d_3	$[M-H]^-$	/	416	140.9	-26	/	/	/
	^{13}C-DON	$[M+CH_3COO]^-$	/	370.1	310.1	-16	/	/	/
	^{13}C-D3G	$[M+CH_3COO]^-$	/	538.2	447.3	-30	/	/	/
	^{13}C-NIV	$[M+CH_3COO]^-$	/	386.1	295.1	-22	/	/	/
	^{13}C-3AcDON	$[M+CH_3COO]^-$	/	414.4	354.2	-12	/	/	/
	^{13}C-15AcDON	$[M+CH_3COO]^-$	/	414.4	354.2	-10	/	/	/

Table S4

Analytes	R^2	LOD (μg/L)	LLOQ (μg/L)	Spiked level (μg/L)	Recovery (n= 3, %)		Matrix effect (n= 3, %)		Intra-day(n= 6, %)		Inter-day (n = 36, %)	
					Accuracy (%)	RSD%	Accuracy (%)	RSD%	Accuracy (%)	RSD%	Accuracy (%)	RSD%
AFB_1	0.9963	0.01	0.02	0.02	92.9	7.3	98.6	2.7	88.6	11.3	100.6	7.5
				1	94.5	2.5	97.2	8.3	99.6	10.8	95.2	4.4
				1.6	98.5	7.2	101.2	11.3	91.7	2.1	95.9	12.7
AFB_2	0.9982	0.01	0.02	0.02	113.0	7.1	106.4	2.8	92.6	8.5	90.2	9.8
				1	107.6	5.6	101.0	8.1	84.9	3.9	98.8	5.8
				1.6	110.8	3.3	104.2	5.1	93.4	10.4	109.1	4.2
AFG_1	0.9971	0.005	0.01	0.01	85.9	10.0	78.1	11.6	95.8	9.3	99.3	3.6
				0.5	90.1	8.5	82.3	9.0	89.4	2.1	112.6	4.5
				0.8	90.1	6.0	82.3	4.1	105.2	9.4	94.7	6.4
AFG_2	0.998	0.01	0.02	0.02	92.0	10.0	92.5	4.0	90.3	5.7	96.6	4.4
				1	101.7	3.7	93.7	8.0	92.5	4.1	93.4	11.7
				1.6	98.3	2.9	90.3	4.8	98.1	6.5	108.5	5.2
AFM_1	0.9992	0.002	0.005	0.005	93.2	3.0	81.3	3.5	102.9	7.5	112.6	5.6
				0.25	96.5	7.7	84.6	3.4	85.8	9.6	110.5	3.5
				0.4	91.4	6.0	79.5	5.0	92.4	8.2	105.3	5.5
AFM_2	0.998	0.002	0.005	0.005	94.3	3.8	90.4	12.2	86.8	2.8	86.1	4.4
				0.25	94.2	7.4	90.3	8.0	102.7	8.5	101.1	3.9
				0.4	91.6	3.8	87.7	4.0	97.9	3.6	106.1	4.3
FB_1	0.9993	0.04	0.1	0.1	97.9	2.4	72.2	11.0	112.3	5.9	102.9	9.5
				5	89.7	4.0	64.0	8.0	90.8	5.6	98.7	10.7
				8	93.9	2.2	68.2	3.0	89.9	11.3	97.3	8.6
FB_2	0.9985	0.04	0.1	0.1	98.6	10.2	67.1	6.0	101.9	2.8	98.8	9.7
				5	96.9	2.3	65.4	4.0	89.6	3.5	103.6	9.4
				8	92.5	9.0	70.0	5.0	102.4	3.4	110.8	7.4
FB_3	0.9977	0.02	0.05	0.05	101.3	7.6	67.1	6.8	104.2	3.1	98.5	3.3
				2.5	109.4	4.5	75.2	6.8	94.8	2.6	93.1	11.2
				4	101.6	1.7	67.4	7.8	106.3	9.9	89.6	9.2

Analytes	R^2	LOD （μg/L）	LLOQ （μg/L）	Spiked level （μg/L）	Recovery (n= 3, %)		Matrix effect (n= 3, %)		Intra-day(n= 6, %)		Inter-day (n = 36, %)	
					Accuracy (%)	RSD%	Accuracy (%)	RSD%	Accuracy (%)	RSD%	Accuracy (%)	RSD%
OTA	0.9995	0.002	0.005	0.005	80.6	8.0	69.6	6.7	98.5	9.4	100.6	5.1
				0.25	87.0	6.3	69.7	4.6	89.7	1.8	83.9	9.3
				0.4	84.0	3.2	70.4	3.4	93.2	9.4	106.9	10.3
OTB	0.9978	0.002	0.005	0.005	84.5	9.0	80.8	12.0	97.3	8.1	103.1	7.3
				0.25	87.5	8.4	83.8	11.4	92.1	9.3	115.3	13.1
				0.4	82.8	2.0	79.1	4.3	102.2	11.5	107.7	5.2
HT2	0.9961	0.05	0.1	0.1	110.6	3.7	80.1	11.0	98.4	4.4	100.7	9.6
				5	102.5	8.7	72.0	9.0	87.9	2.8	88.3	4.6
				8	109.0	7.2	78.5	4.6	98.9	8.4	113.7	3.9
T2	0.9989	0.005	0.01	0.01	94.0	4.7	105.0	10.0	91.2	9.8	111.5	12.4
				0.5	106.2	7.6	100.3	4.0	89.4	4.8	90.4	5.4
				0.8	103.6	7.4	97.7	8.2	104.1	9.4	98.9	13.8
T2(OH) 3	0.9953	0.01	0.02	0.02	94.1	6.7	69.1	9.0	98.9	6.5	104.9	8.7
				1	95.6	4.1	70.6	6.9	84.2	7.6	103.2	7.4
				1.6	94.4	8.0	69.4	7.0	95.4	9.8	108.9	3.7
Acl	0.991	0.001	0.002	0.002	88.4	5.6	88.9	6.0	84.8	10.1	89.3	6.2
				0.1	81.2	1.9	81.7	3.0	92.6	8.6	86.8	4.6
				0.16	83.8	3.3	84.3	4.6	87.8	2.8	80.2	12.2
Ecl	0.9973	0.001	0.002	0.002	59.6	9.9	65.2	9.0	66.4	2.5	77.8	8.9
				0.1	63.9	5.0	64.5	3.0	78.7	9.5	79.9	7.9
				0.16	60.0	2.0	69.6	4.0	72.8	2.6	75.2	3.2
Ergine	0.9978	0.002	0.005	0.005	79.0	7.7	68.1	5.0	86.5	10.1	88.3	10.4
				0.25	73.4	7.3	62.5	11.0	91.1	2.9	81.7	7.5
				0.4	81.5	3.0	70.6	9.0	85.2	2.2	80.8	5.3
Eco	0.995	0.02	0.04	0.04	93.2	11.0	98.5	2.2	98.4	7.8	96.5	3.1
				2	117.3	4.0	102.6	7.4	90.7	4.3	104.3	4.7
				3.2	113.5	7.5	98.8	10.0	99.4	8.4	108.9	5.2
Econ	0.9937	0.002	0.005	0.005	95.0	11.0	76.4	11.1	105.2	4.3	101.3	3.6
				0.25	91.0	1.8	82.2	8.0	87.4	8.1	96.9	11.2

Analytes	R^2	LOD (μg/L)	LLOQ (μg/L)	Spiked level (μg/L)	Recovery (n= 3, %)		Matrix effect (n= 3, %)		Intra-day(n= 6, %)		Inter-day (n = 36, %)	
					Accuracy (%)	RSD%	Accuracy (%)	RSD%	Accuracy (%)	RSD%	Accuracy (%)	RSD%
				0.4	96.2	2.8	77.6	7.0	91.6	2.4	89.3	9.1
				0.02	91.0	6.0	81.3	8.6	89.6	10.2	97.9	5.2
Ecr	0.9992	0.01	0.02	1	111.0	3.0	89.8	3.8	88.1	1.9	88.8	7.3
				1.6	103.8	4.5	82.6	6.8	91.9	8.4	106.4	3.4
				0.02	95.1	10.0	83.4	11.3	91.2	3.8	96.4	8.1
Ecrn	0.9984	0.01	0.02	1	96.3	7.4	84.6	8.7	88.2	3.7	98.3	4.7
				1.6	93.6	4.1	81.9	3.7	84.7	6.5	105.3	9.7
				0.01	86.0	7.0	77.8	6.5	89.4	11.3	108.8	9.7
DH-Ecr	0.9986	0.005	0.01	0.5	94.2	5.6	86.0	2.5	95.9	9.3	110.8	5.7
				0.8	91.5	4.0	83.3	5.7	93.1	11.5	88.5	6.5
				0.01	118.9	1.7	112.8	4.5	93.5	5.8	109.9	4.5
Ek	0.9933	0.005	0.01	0.5	118.2	4.0	112.1	3.0	103.1	2.6	100.1	3.3
				0.8	113.9	7.9	107.8	6.6	90.3	5.1	103.3	12.7
				0.01	110.0	7.7	97.6	10.8	93.7	10.1	87.3	6.7
Ekn	0.9967	0.005	0.01	0.5	116.3	4.5	96.9	6.0	105.7	6.5	100.1	4.7
				0.8	118.3	1.9	98.9	2.3	89.2	6.8	108.4	4.1
				0.002	85.5	4.4	82.9	8.0	97.3	5.2	90.3	11.8
Em	0.9963	0.001	0.002	0.1	86.7	4.7	84.1	5.0	96.4	4.2	110.6	11.7
				0.16	89.1	3.0	86.5	4.8	101.9	3.7	89.7	5.7
				0.01	89.3	9.1	90.7	10.0	104.5	9.1	109.1	7.8
Emn	0.9932	0.005	0.01	0.5	97.5	5.0	98.9	4.0	93.3	3.8	102.8	11.2
				0.8	94.4	3.0	95.8	6.3	94.5	2.1	91.2	6.7
				0.005	100.3	10.3	91.3	9.3	98.6	11.1	88.4	10.7
Etn	0.9961	0.002	0.005	0.25	95.6	9.0	86.6	5.0	90.3	6.4	85.6	5.6
				0.4	92.6	5.7	83.6	7.3	108.5	2.2	94.1	9.2
				0.002	91.6	8.5	87.1	8.0	104.5	11.4	97.8	6.4
Esn	0.9976	0.001	0.002	0.1	92.9	7.0	88.4	8.0	105.8	10.1	116.9	3.5
				0.16	91.1	2.2	86.6	4.0	97.7	10.7	101.1	6.1
Et	0.9935	0.02	0.05	0.05	92.0	3.0	77.0	6.0	99.2	5.5	105.3	8.7

Analytes	R^2	LOD（μg/L）	LLOQ（μg/L）	Spiked level（μg/L）	Recovery (n= 3, %)		Matrix effect (n= 3, %)		Intra-day(n= 6, %)		Inter-day (n = 36, %)	
					Accuracy (%)	RSD%	Accuracy (%)	RSD%	Accuracy (%)	RSD%	Accuracy (%)	RSD%
				2.5	104.2	2.5	85.7	4.2	104.6	2.8	111.6	9.2
				4	95.3	1.7	84.0	3.0	84.9	2.5	93.8	8.7
				0.05	80.0	3.2	88.7	8.3	77.8	11.2	68.6	8.2
DH-LYS	0.9962	0.01	0.05	2.5	83.0	3.2	80.2	4.1	75.2	9.9	79.8	6.6
				4	79.5	9.0	80.9	3.6	78.2	3.6	78.7	3.4
				0.02	89.2	8.3	96.1	10.8	93.2	3.3	84.4	4.7
15AS	0.9931	0.01	0.02	1	95.2	3.8	102.1	5.5	95.1	9.1	110.9	8.5
				1.6	98.1	6.0	105.0	6.0	97.8	9.2	96.8	3.8
				0.05	137.1	12.0	139.3	5.3	105.6	2.2	109.1	8.7
DAS	0.9985	0.02	0.05	2.5	145.5	3.8	147.7	7.1	98.6	9.6	113.5	6.7
				4	143.4	2.5	155.6	5.7	93.2	9.5	99.2	7.9
				0.01	83.9	7.5	79.7	4.8	104.9	6.7	99.5	5.3
NEO	0.9954	0.005	0.01	0.5	84.4	7.7	80.2	11.5	91.9	5.2	111.8	4.6
				0.8	85.0	9.2	80.8	6.4	90.5	9.3	102.7	11.4
				0.1	104.6	11.0	99.3	5.5	102.1	8.5	86.9	8.1
CIT	0.9953	0.04	0.1	5	110.9	6.0	105.6	7.0	94.6	2.3	97.2	4.1
				8	111.0	3.0	105.7	2.1	89.4	4.2	89.3	10.3
				0.1	74.0	11.0	72.5	6.0	72.5	8.2	78.9	7.8
PCA	0.9976	0.04	0.1	5	81.0	9.0	79.0	2.9	76.2	9.6	81.8	10.2
				8	78.9	8.4	76.9	3.0	77.4	11.1	80.2	9.2
				0.01	68.0	10.0	66.0	9.9	98.7	11.3	101.3	8.5
RC	0.997	0.005	0.01	0.5	78.0	2.2	69.0	4.5	93.7	7.6	115.1	4.6
				0.8	75.0	6.4	65.6	6.0	91.6	2.9	109.5	4.1
				0.002	81.0	12.0	84.5	11.0	85.7	8.6	95.8	4.1
CPA	0.993	0.001	0.002	0.1	87.9	4.0	85.1	2.8	84.3	6.1	101.1	3.8
				0.16	87.0	1.4	89.0	6.1	87.3	3.1	90.6	3.6
				0.002	84.5	10.5	64.7	8.0	94.8	8.3	96.2	11.9
STG	0.9973	0.001	0.002	0.1	82.8	2.1	70.0	5.7	103.7	11.7	95.9	3.3
				0.16	82.7	2.9	68.0	5.0	99.1	9.5	102.1	6.1

Analytes	R^2	LOD (μg/L)	LLOQ (μg/L)	Spiked level (μg/L)	Recovery (n= 3, %)		Matrix effect (n= 3, %)		Intra-day(n= 6, %)		Inter-day (n = 36, %)	
					Accuracy (%)	RSD%	Accuracy (%)	RSD%	Accuracy (%)	RSD%	Accuracy (%)	RSD%
GLIO	0.9973	0.1	0.5	0.5	94.3	10.0	109.2	7.3	101.2	5.9	106.4	8.9
				25	98.4	2.0	113.3	4.5	98.5	11.9	101.4	3.5
				40	95.8	6.3	110.7	2.6	94.8	3.8	110.4	7.6
MPA	0.9968	0.005	0.01	0.01	109.7	10.0	94.6	11.6	91.8	4.5	112.4	7.5
				0.5	112.3	6.3	97.2	4.4	92.5	10.7	85.5	3.3
				0.8	108.3	1.5	93.2	3.0	89.2	2.5	107.8	10.5
BEA	0.9902	0.005	0.01	0.01	133.8	2.3	127.7	12.3	112.6	8.7	111.6	3.5
				0.5	128.9	2.5	122.4	8.0	101.7	10.3	109.7	6.8
				0.8	131.7	12.0	125.2	7.8	116.6	7.9	109.3	7.1
EnA	0.9913	0.005	0.01	0.01	146.4	6.0	96.0	8.8	129.8	7.6	125.8	5.7
				0.5	144.9	10.8	104.4	11.5	117.1	6.2	117.1	8.9
				0.8	144.2	3.6	103.7	9.0	125.7	7.4	122.4	6.9
EnA_1	0.9907	0.001	0.002	0.002	129.0	3.0	98.5	9.0	109.7	5.6	111.6	5.8
				0.1	129.6	9.0	96.2	6.0	110.3	10.4	108.5	7.4
				0.16	132.0	3.0	95.8	3.0	115.9	11.6	109.2	3.3
EnB	0.9906	0.001	0.002	0.002	106.8	8.0	80.0	13.0	98.6	4.9	92.2	7.7
				0.1	105.6	1.6	78.8	6.6	95.4	9.3	98.8	8.6
				0.16	107.7	2.0	80.9	4.0	93.7	3.7	93.6	7.4
EnB_1	0.9913	0.001	0.002	0.002	121.1	1.9	80.7	11.0	107.3	6.5	107.6	4.7
				0.1	124.0	2.7	83.6	7.0	112.9	11.5	110.2	6.9
				0.16	127.2	1.8	86.8	5.0	110.1	4.8	109.6	8.6
ZAN	0.9975	0.02	0.05	0.05	83.6	4.9	74.8	12.0	84.7	9.6	99.5	5.1
				2.5	92.0	7.0	83.2	11.0	111.7	7.2	95.2	6.4
				4	91.1	2.3	82.3	5.5	84.9	10.3	102.2	9.4
ZEN	0.9979	0.005	0.02	0.02	88.5	10.0	80.2	8.0	88.3	2.8	113.8	13.7
				1	89.4	1.5	81.1	9.0	86.7	5.4	95.4	9.7
				1.6	82.5	4.0	74.2	4.0	107.7	9.3	100.1	6.5
α-ZEL	0.998	0.02	0.05	0.05	83.0	7.9	87.0	9.2	84.1	4.7	84.8	5.1
				2.5	92.2	1.6	86.3	4.0	107.5	10.2	83.4	5.1

Analytes	R^2	LOD （μg/L）	LLOQ （μg/L）	Spiked level （μg/L）	Recovery (n= 3, %)		Matrix effect (n= 3, %)		Intra-day(n= 6, %)		Inter-day (n = 36, %)	
					Accuracy (%)	RSD%	Accuracy (%)	RSD%	Accuracy (%)	RSD%	Accuracy (%)	RSD%
				4	86.4	8.1	80.5	5.1	93.2	5.9	100.2	3.9
				0.05	78.7	5.8	80.5	7.9	100.8	3.4	111.8	4.6
β-ZEL	0.9968	0.02	0.05	2.5	81.0	10.0	82.8	10.4	85.5	9.5	85.9	10.7
				4	79.3	1.9	81.1	8.9	96.9	4.7	93.4	7.5
				0.04	77.5	4.8	71.9	8.0	84.8	10.1	106.8	8.4
α-ZAL	0.9974	0.02	0.04	2	82.7	9.0	77.1	2.4	95.6	2.9	101.5	3.1
				3.2	81.5	6.0	75.9	6.0	83.1	2.5	92.6	6.9
				0.05	80.0	9.9	71.0	7.0	106.5	9.8	113.9	8.5
β-ZAL	0.9978	0.02	0.05	2.5	83.5	5.7	75.8	4.0	102.7	4.5	93.2	11.1
				4	88.0	7.8	72.2	3.8	110.2	6.2	117.2	8.3
				0.04	74.1	8.4	64.9	10.9	93.4	4.1	85.5	12.6
AOH	0.9979	0.02	0.04	2	73.0	8.0	65.1	6.4	97.5	11.2	109.4	4.4
				3.2	74.5	4.0	66.6	5.0	106.2	2.1	117.7	3.9
				0.01	79.5	6.7	75.7	10.3	96.9	4.4	93.3	9.3
AME	0.9973	0.003	0.01	0.5	86.6	3.6	82.8	4.0	85.8	6.4	103.2	4.8
				0.8	81.4	5.9	77.6	6.9	93.4	2.7	93.4	4.6
				0.08	71.9	7.0	75.1	4.5	86.8	2.7	90.4	13.5
ALT	0.9972	0.04	0.08	4	70.2	9.6	73.4	9.0	91.3	6.4	96.3	4.3
				6.4	77.6	5.9	80.8	3.0	109.1	9.2	89.5	12.5
				0.1	92.0	12.2	91.6	5.9	100.5	5.8	105.8	5.8
TeA	0.9984	0.05	0.1	5	105.1	9.5	94.4	8.0	88.5	10.3	90.6	5.3
				8	105.9	3.0	95.2	4.7	90.5	11.5	99.2	8.9
				0.02	86.6	8.0	85.1	4.6	89.6	2.9	101.5	11.9
AXT I	0.9999	0.01	0.02	1	92.6	6.0	91.1	5.2	103.4	3.7	86.2	10.2
				1.6	92.0	3.9	90.5	3.0	109.6	9.4	109.3	13.3
				0.04	76.1	11.0	79.2	9.0	93.8	11.7	108.9	11.6
TEN	0.9982	0.02	0.04	2	76.0	5.7	79.1	7.6	83.1	6.2	104.9	7.2
				3.2	69.4	9.3	72.5	3.0	88.2	6.6	83.9	7.7
MON	0.995	0.2	0.4	0.4	76.4	6.0	70.5	9.5	102.8	4.7	102.7	4.5

Analytes	R^2	LOD（μg/L）	LLOQ（μg/L）	Spiked level（μg/L）	Recovery (n= 3, %)		Matrix effect (n= 3, %)		Intra-day(n= 6, %)		Inter-day (n = 36, %)	
					Accuracy (%)	RSD%	Accuracy (%)	RSD%	Accuracy (%)	RSD%	Accuracy (%)	RSD%
				20	72.3	3.7	66.4	5.5	93.6	3.9	101.7	11.4
				32	77.0	8.0	71.1	4.3	93.5	4.2	87.4	5.4
				0.02	91.4	9.0	96.2	10.0	88.7	5.6	87.9	6.7
OTα	0.9955	0.01	0.02	1	93.7	3.2	98.5	3.0	86.8	11.1	91.9	10.9
				1.6	95.9	4.3	100.7	5.0	98.3	8.2	107.7	13.3
				1	104.2	6.5	64.7	2.3	86.5	7.3	90.8	10.3
PAT	0.9952	0.5	1	50	108.7	5.7	79.2	8.2	89.7	3.9	86.2	4.5
				80	103.0	5.4	68.5	4.0	94.2	10.6	99.9	7.2
				0.04	83.0	3.8	70.5	9.0	82.7	6.4	106.9	11.6
D3G	0.9992	0.02	0.04	2	87.3	7.1	74.8	11.4	96.8	2.3	108.7	5.8
				3.2	86.2	3.5	73.7	3.1	90.9	10.2	95.2	7.4
				0.4	85.3	5.5	80.0	10.0	103.3	3.6	93.4	9.3
DON	0.998	0.2	0.4	20	82.0	6.0	86.4	2.8	100.4	3.3	109.1	6.3
				32	84.5	7.9	88.9	7.8	110.7	8.3	97.7	11.4
				0.2	75.1	10.2	70.9	5.1	95.7	8.2	92.2	10.5
FusX	0.9974	0.05	0.2	10	72.8	2.9	68.6	9.7	107.5	11.7	108.1	12.1
				16	73.5	5.0	69.3	5.8	96.4	2.8	83.6	6.3
				0.2	89.1	4.5	69.0	9.0	108.8	5.4	98.6	7.7
NIV	0.9989	0.1	0.2	10	97.2	3.3	67.1	5.9	83.7	2.6	101.6	12.9
				16	88.2	8.9	68.1	5.1	98.9	5.2	84.8	11.1
				0.4	82.0	5.0	73.0	6.2	96.3	8.5	86.1	9.2
DOM	0.9974	0.2	0.4	20	87.7	7.3	80.7	3.2	99.9	3.6	108.6	6.6
				32	92.0	4.6	74.6	5.0	89.5	10.6	93.8	6.7
				0.2	92.8	2.6	85.9	4.6	102.5	5.2	85.4	10.1
3AcDON	0.9971	0.05	0.2	10	91.5	2.8	84.6	2.8	89.3	8.8	90.6	3.5
				16	90.4	3.1	83.5	10.1	88.2	6.2	88.9	3.1
				0.8	80.0	11.0	85.2	7.1	84.5	2.4	94.5	8.5
15AcDON	0.996	0.4	0.8	40	89.5	5.0	85.1	2.3	88.1	4.8	90.4	6.2
				64	91.4	2.0	87.0	5.5	94.9	5.4	98.2	7.1

Table S5

Analytes	Spiked level （μg/L）	0h	4℃ for 3 h	4℃ for 6 h	4℃ for 1day	4℃ for 3days	4℃ for 5days	25℃ for 3 h	25℃ for 6 h	25℃ for 1day	25℃ for 3days	25℃ for 5days
**AFB$_1$**	0.04	95.1	90.7	91.3	78.8	68.1	56	88.2	75.2	70.9	57.4	44.1
	2	94.7	96.4	94.3	83.1	73.6	61.9	90.9	89.3	76.4	69.7	50.8
**AFG$_1$**	0.02	86	84.7	83.4	75	52.4	23.2	86.1	70.7	64	44.5	21.6
	1	90.3	91.3	81.5	80	60.2	29.9	85.1	82.9	64.6	50.9	26.6
**AFG$_2$**	0.04	92.8	91.1	90.7	85.6	71	41.4	92.1	78.5	75.5	62.6	40.3
	2	103.9	102.5	98.5	91.6	77.2	54.6	102.7	96.2	87.6	74.1	62.5
**AFM$_1$**	0.04	92.6	91.7	90.9	81.9	66.8	43.9	94.1	79.7	76	57.8	30.8
	2	96.2	95.3	94.4	81.4	77.8	54.7	93.5	90.7	79.4	66.3	45.2
**CIT**	0.2	103.6	103.1	102.5	73.2	63.2	34.6	103.8	90.3	71.3	56.1	26.8
	10	113.1	110.3	108.3	90	76.2	58.3	111.9	107.7	74.5	63.1	49
**HT2**	0.04	111	111.9	102	80.7	72.8	57.4	108.9	106.4	78	67.8	54.9
	2	103.5	104.2	99.4	92.5	86.6	71.6	97.1	87.2	79.9	60.5	58.8
**T2**	0.04	94.8	94.1	93.1	83.4	67.3	27.2	88.9	85.7	76.2	56	22.9
	2	107.7	106.8	103.1	90.1	77.3	28.8	100.6	98.5	73.1	51.6	25.1
**T2(OH) 3**	0.04	96.1	93.1	90.8	79.6	64.3	49.1	94	88.4	65.7	60.7	40.9
	2	95.5	95	91.4	88.6	78	60.8	95	89.4	85.8	69.6	59.4
**DON**	0.04	84.9	88.3	86.7	70.2	66.6	52	79.7	75.3	63.3	55.5	33.9
	2	81.2	87.8	81.9	75.2	71.1	68.4	80.5	73.8	70.7	68.8	31.1
**DOM**	0.8	82.9	82	83.1	76.1	70	63.5	78.2	77	63.9	58.4	30.8
	40	88.5	87.3	88.7	88.4	83.2	71.4	83.8	81.7	72.8	61.8	36.7
**AFB$_2$**	0.04	113.7	110.8	111.2	100.8	99.3	86.8	114.2	110.6	96.8	85.3	77.6
	2	109.6	106.3	107.8	90.1	87.4	75.9	102.7	99.9	91.2	85.5	74.4
**AFM$_2$**	0.04	93.7	93.9	91.1	87.9	72.2	56.4	91.3	88.9	85.3	72.1	49.7
	2	96.3	95.1	95.9	91	87.9	63.8	90.7	95.2	82.1	72.6	57.4
**FB$_1$**	0.02	96.9	95.6	96.2	91.9	83.3	64.1	94	97.5	92.8	81.4	60.6
	1	100.7	96.3	94.4	90.6	87.5	71	87.8	90.8	98.2	80	70.2
**FB2**	0.04	97.6	94.6	99.1	82.7	72.7	58	95.2	92.4	69.9	62.1	59.6
	2	95.7	93.7	94.3	90.7	85.9	72.2	92.8	90.5	94.9	83.1	60.3
**FB$_3$**	0.04	102.9	103	98.6	83	80.4	73.5	95.7	101.5	75.1	78.6	65.6
	2	108.1	107.6	110.1	88.7	84.7	80.1	109.3	106.6	82.1	79.8	76.4

Analytes	Spiked level （μg/L）	0h	4℃ for 3 h	4℃ for 6 h	4℃ for 1day	4℃ for 3days	4℃ for 5days	25℃ for 3 h	25℃ for 6 h	25℃ for 1day	25℃ for 3days	25℃ for 5days
OTA	0.04	82	83	81.9	80.6	77	65.5	81	75.7	80.2	68.2	59.1
	2	87	86.8	84.7	84.9	79	76.6	83.1	87.4	83	80.8	65.2
OTB	0.02	83.2	84.1	83.6	93.1	80.7	72.6	78.9	83.9	80.4	77.8	62.9
	1	87.1	88.5	85.7	86	86.9	78.9	88.3	87.5	87.4	76.5	62
Acl	0.02	89.3	86.5	87	79.8	68.9	58.2	86.2	82.6	73.1	64.7	58.7
	1	82.2	83.5	79.3	80.2	80	69.7	76.1	78.6	74.4	71.1	62.1
Ecl	0.04	60.7	60.4	59.7	62.4	60	47.5	59.5	58.6	66.3	50.5	40.3
	2	65.2	65.3	62	61.7	64.1	60.5	58.5	64.7	69.5	56	43.2
Ergine	0.01	80.6	77.8	77.3	80.2	68.6	56.2	79.4	74.5	79.8	57	50.2
	0.5	72.1	75.5	72.7	86.1	70.4	62.3	68.6	73.5	73	64.5	51.3
Eco	0.08	93.3	91.7	90.9	79.7	79.1	74	90.8	90	74.2	72.5	67.6
	4	117.2	120.2	114.5	108.5	102.7	99.3	112.5	118.5	101.1	103.5	90.9
Econ	0.01	94.7	93.1	93.4	91.2	85.6	71.4	96.3	94.2	87.7	77.1	64.4
	0.5	90.5	91.6	91.8	93.4	89.3	78.2	86.3	90.8	93	88.6	70.3
Ecr	0.04	93.2	91.2	88.2	86.5	80.4	72	86.1	92.4	82.4	81.8	67.1
	2	112.8	110.2	108.3	96.2	89.7	81.1	106.4	109.8	90.1	87.8	85.4
Ecrn	0.04	95	96.7	93.5	85.4	79.6	68.1	94.9	87	83.6	78.9	69.4
	2	98.5	98.1	95.7	84.8	89.5	81.2	97.4	91.3	87.5	86.4	72.8
DH-Ecr	0.04	87	86.5	83.2	82.7	80.7	75	84	87.3	79.9	73.7	60.2
	2	94.6	94.1	93.5	87.7	86.9	78.4	93.6	86.1	90	80.9	69.4
Ek	0.02	120.3	120.2	119.5	102.7	97.7	93.8	116.1	118.6	98	99.6	89.8
	1	119.5	117.6	119.4	108	99.9	90.2	119.4	110.7	98.8	95.6	92.4
Ekn	0.04	110.3	109.1	108.1	103.9	108.5	89.8	106	109.7	109.2	90.9	85
	2	116.4	115.9	114.9	110	100.9	91.4	110.5	115.4	109.1	105.9	90.1
Em	0.04	84.4	86.3	82.7	89.2	84.7	79.7	82.9	78.8	82.4	86.5	57.7
	2	86	89.2	88.1	83.7	75.4	72.1	85.3	82.6	79.1	71.4	59.1
Emn	0.04	91.1	92.2	89.9	82.2	86.6	79.9	83.6	84.8	85.9	91.7	66
	2	96.3	96.7	98.6	89.7	88.5	78	94.7	93.4	84.2	91.5	70.2
Etn	0.02	100.6	100.2	99.6	82.1	87.9	81.3	96	99.8	81.7	80.2	70.2
	1	96.6	98.2	95	86.5	85.5	78.5	90	90.7	87.2	86	70.5
Esn	0.04	92.5	94.6	89.7	83.8	78.2	73.6	86.2	83.8	85.5	77.4	66

Analytes	Spiked level （μg/L）	0h	4℃ for 3 h	4℃ for 6 h	4℃ for 1day	4℃ for 3days	4℃ for 5days	25℃ for 3 h	25℃ for 6 h	25℃ for 1day	25℃ for 3days	25℃ for 5days
	2	94.7	95.3	90.3	92	87.4	80.1	91.4	92.2	86	87.8	69.2
**Et**	0.04	91	93.8	89	81.6	79.6	71.4	86.8	83.1	79.7	82.5	60.9
	2	105.7	105.6	104.3	92.9	84.8	77.9	99.3	99.7	92.6	88.2	79.5
**DH-LYS**	0.04	78.9	79.3	76.8	77.9	76.7	68	76.5	79.6	77.9	71.5	52.3
	2	84	85.1	82.8	72.8	72.3	61	84.3	77.2	70.4	74	58.1
**15AS**	0.02	88.4	91.4	86.8	81.7	79.2	65.1	86.3	87.5	71.9	69.1	56.9
	1	94.8	97.5	94.3	88.7	83.2	74	89.8	89.5	87.9	89.9	68.2
**DAS**	0.04	137.1	137.8	133.8	122.7	128.9	118.2	132.4	133	112.9	114.2	106.7
	2	146	141	143.4	138.5	134.1	126.1	142	142.9	128.8	123.7	117.4
**NEO**	0.02	85.9	84.4	80.9	72.2	77.5	63.9	81.1	80.2	75.1	67.6	59.1
	1	83.2	82.3	80.3	83.6	79.5	71.2	79.7	77.1	80.2	75.7	57.7
**PCA**	0.2	74.8	72.2	71.9	69	63.7	55	69.2	69.7	61.2	58.5	46.7
	10	81	84	82.3	73.4	69.5	62.2	77.9	76.8	68	66.6	51.1
**RC**	0.02	68.5	70.2	65.8	72.9	65.8	59.4	69.5	66.8	62.6	59.9	41.9
	1	79.5	79	76.9	76.8	70.7	62.7	72.8	79.2	75.1	71.1	48.3
**CPA**	0.04	83	83.1	79.7	72	71.1	66.4	81.2	80.8	70.4	67.1	53.5
	2	89.9	86.3	84.9	79.1	79.5	75.7	83.5	84	80.2	73.5	62.8
**STG**	0.04	86.6	82.8	81.9	73.3	71.8	62.4	83.1	79.1	70.5	64.6	53.7
	2	81.8	82.1	84.2	80.1	80.1	74.6	84.3	78.6	78	71.7	49.2
**GLIO**	0.02	95.5	92.3	94.3	86	84.2	70	93.9	92	82.4	73.4	63.3
	1	97.6	101.1	97.9	87	81.1	71.7	99.8	89.8	85.1	76.9	69
**MPA**	0.04	111.1	108.1	108.4	93.6	79.2	73.2	110.3	104.3	83.4	81	73.5
	2	111.2	115.4	109.9	101.7	89.7	77.8	110.9	107.5	94	83.5	71.5
**BEA**	0.04	132.9	134.3	134.3	124	114.4	105.2	128.6	127.1	114.1	111.2	106.6
	2	129.5	131.3	129.3	123.1	119.2	110.5	127.9	127.9	115.9	111.5	95
**EnA**	0.04	145.8	146.7	146.1	145.8	133.7	129.6	147.2	147.5	141.4	139.1	114.5
	2	146.1	144.6	142.4	138.4	135.6	123.5	141.2	145.7	130.3	123.2	120.7
**EnA_1**	0.02	130.8	127.7	130.3	122.2	125.2	119.1	125.8	129.7	114.4	105.9	102.7
	1	131.6	128.8	130.6	124.5	121.4	118.3	126.3	125	117	110.5	105.4
**EnB**	0.04	105.8	107.7	105.1	100.8	99.8	89.3	106.4	98.9	97.6	91.3	72
	2	107.2	105.3	103.7	106.7	101.7	91	102.5	99	97.7	96.1	74

Analytes	Spiked level （μg/L）	0h	4℃ for 3 h	4℃ for 6 h	4℃ for 1day	4℃ for 3days	4℃ for 5days	25℃ for 3 h	25℃ for 6 h	25℃ for 1day	25℃ for 3days	25℃ for 5days
**EnB_1**	0.04	123.3	119	121	114.6	113.2	104.4	119	117.4	108.4	98	96.4
	2	125.1	123.7	122.5	117.3	111.9	106.3	124	124	112.8	106.1	99.2
**ZAN**	0.04	85.8	85.2	82.9	78.3	80.4	72	78.5	77.5	78.8	71.4	58
	2	91.9	91.3	91.2	86.5	83.9	88.2	87	88.2	85.9	75.9	65.7
**ZEN**	0.02	90.7	86.8	86.8	89.6	88.6	85	82.8	85.5	83.8	75.7	68.6
	1	91.1	90.6	86.7	86.6	84.3	82.8	86.2	85.9	85.3	76.9	69.2
**α-ZEL**	0.04	82.1	80.7	80.8	77.9	76.8	68.2	80.1	76.9	77.3	71.5	60.7
	2	93.5	91.1	91.9	89.4	85.8	76.9	89.7	88.7	86.6	73	68.8
**β-ZEL**	0.1	77.7	77.1	76.4	76.2	72.5	62	74	74.6	73.8	64.5	56.9
	5	81.6	83.3	78.4	77.4	75.1	65.1	77.4	73.8	76.3	69.1	54.1
**α-ZAL**	0.08	79.1	76.9	74.8	70.8	69.1	66.2	77.5	70.6	70.8	65.2	53.6
	4	81.9	83.5	81.7	75	76	71.6	78.4	75.4	72.9	67.1	60.6
**β-ZAL**	0.1	80	82.6	76.9	80.7	71.5	63.4	77.2	77.2	73.2	71.6	55.7
	5	83.8	84.1	84.5	81.3	78.3	68.9	84.7	81.5	80.2	75.1	60.6
**AOH**	0.08	76.3	76.4	71.6	70.6	70.5	61.6	72.7	71.5	70.5	68.4	56.1
	4	74.2	74.4	72.3	71	66.3	57.3	71.7	71.5	69.6	69.9	47.9
**AME**	0.04	81.1	78	77.5	90.7	74.5	65.7	80.9	74.1	87.4	69.5	53.7
	2	85.8	86.1	86.7	88.9	79.2	68.2	84.4	80.9	80.2	72.3	62.7
**ALT**	0.04	71.7	73.5	72.2	70.6	66.1	63.1	66.6	67.2	79.2	68.4	46.8
	2	72.1	73.2	67.6	90.4	76.1	64.6	66.8	65	70.5	61.9	50.3
**TeA**	0.02	92	92.3	90.1	81.4	78.7	69.4	87.4	86	81.5	80.5	71.1
	1	104.3	104.7	106.8	90.5	89.5	88.3	104.9	103.1	90.3	82.7	81.7
**AXT I**	0.04	88.1	86.6	85	81.6	81.4	79.5	86.3	82.6	84.8	86.4	60.7
	2	93.3	92.5	89.8	89.7	86.3	81.3	90.7	94	83.9	82.4	70.2
**TEN**	0.04	76.6	76.7	76.9	63.8	66.7	58	72.5	67.8	67.6	70.4	56.1
	2	77.5	76.8	75.3	72.1	66.5	55.7	72.3	73.9	68.4	67.5	56.9
**MON**	0.04	76.3	75.2	74.3	70.8	69.2	58.2	74.5	69	70.3	68.2	51.4
	2	73.7	72.6	70.6	72.7	70	60.6	71.2	70.9	71.5	66.2	48
**OTα**	0.02	91.4	92	91	85.5	81.4	75.8	90.5	85	82.7	70.6	65.3
	1	94.7	94.4	91.5	88.6	89.3	74	88.7	87.9	87.6	76.8	70.5
**PAT**	0.04	103.9	103.3	101.7	99.6	93.3	86.8	102.5	103	96.8	88.4	81.7

Analytes	Spiked level （μg/L）	0h	4°C for 3 h	4°C for 6 h	4°C for 1day	4°C for 3days	4°C for 5days	25°C for 3 h	25°C for 6 h	25°C for 1day	25°C for 3days	25°C for 5days
	2	107.9	109.1	107.6	105	98.4	88.6	104.4	104.2	107.7	97.7	87
**D3G**	0.04	84.3	85.7	84	82.6	75.8	65.1	79.4	71.7	61.8	51.7	39.2
	2	87.7	86.1	84.8	84.3	79.4	73.9	82.7	72.5	70	67	54.9
**FusX**	0.02	76.4	75.4	76.1	72.1	68.1	58.7	70	67.3	67.5	54.8	34.8
	1	74.6	70.8	69.8	72	66.7	57.1	71	70.4	69	52.9	44.5
**NIV**	0.04	90.2	88.5	86.4	84.1	79.7	63.4	85.4	83.5	72.1	67.7	49.4
	2	98.4	97.3	97.8	91.1	82.6	74.9	97.9	90.2	80	72.6	65.9
**3AcDON**	0.4	93	94.2	93.3	93.3	72	63.2	93.2	85.7	79.1	66.6	56.1
	20	90.8	94.4	91	90.9	84.4	72.9	91.3	83.8	80.5	69	67
**15AcDON**	1.6	88.9	86.3	80.7	76.2	74.8	64.2	83.5	79.8	63.8	56.8	45.3
	80	90.6	90.8	86.8	86.4	81.8	69.4	90.7	84.9	80.2	78	68.7

Table S6

Analyte	The lowest LODs in previous studies(μg/L)	The LODs in this study(μg/L)	Multiple of LODs reduction	Analyte	The lowest LODs in previous studies(μg/L)	The LODs in this study(μg/L)	Multiple of LODs reduction
AFB1	0.0012	0.01	0.1	EnB1	0.0044	0.001	4.4
AFB2	0.0013	0.01	0.1	FB1	0.2	0.04	5.0
EnA	0.0016	0.005	0.3	EnA1	0.0055	0.001	5.5
AFG2	0.0037	0.01	0.4	DOM	1.35	0.2	6.8
AFG1	0.0021	0.005	0.4	AOH	0.142	0.02	7.1
PAT	0.35	0.5	0.7	DAS	0.15	0.02	7.5
AFM1	0.0017	0.002	0.9	FB2	0.39	0.04	9.8
CIT	0.04	0.04	1.0	ZEN	0.05	0.005	10
EnB	0.0012	0.001	1.2	3-AcDON	0.7	0.05	14
DON	0.263	0.2	1.3	STG	0.05	0.002	25
OTα	0.014	0.01	1.4	HT2	1.344	0.05	27
ZAN	0.03	0.02	1.5	NEO	0.18	0.005	36
BEA	0.014	0.005	2.8	FUS-X	1.95	0.05	39
15-AcDON	1.2	0.4	3.0	T2	0.2	0.005	40
OTA	0.006	0.002	3.0	AME	0.146	0.003	49
α-ZAL	0.07	0.02	3.5	GLIO	5.5	0.1	55
β-ZAL	0.07	0.02	3.5	D3G	1.287	0.02	64
β-ZEL	0.07	0.02	3.5	NIV	9.1	0.1	91
ALT	0.147	0.04	3.7	OTB	0.4	0.002	200

Table S7

Mycotoxins		Total						Male Infertility						Control						Detections
		Detections	Concentration (μg/L)					Detections	Concentration (μg/L)					Detections	Concentration(μg/L)					*P* value*
		n (%)	Min	Q1	Median	Q3	Max	n (%)	Min	Q1	Median	Q3	Max	n (%)	Min	Q1	Median	Q3	Max	
**Traditional mycotoxins**																				
Aflatoxins	Total	36 (19.6)	0.003	0.014	0.019	0.03	0.145	22 (24.7)	0.003	0.015	0.02	0.031	0.145	14 (14.7)	0.006	0.011	0.017	0.03	0.048	0.088
	AFB_2	25 (13.6)	0.011	0.015	0.018	0.031	0.055	16 (18.0)	0.012	0.015	0.018	0.028	0.055	9 (9.5)	0.011	0.016	0.018	0.031	0.048	0.093
	AFG_1	7 (3.8)	0.006	0.009	0.015	0.02	0.029	4 (4.5)	0.009	0.012	0.017	0.024	0.029	3 (3.2)	0.006	0.006	0.009	0.016	0.016	0.714
	AFG_2	5 (2.7)	0.01	0.019	0.02	0.03	0.145	4 (4.5)	0.01	0.015	0.019	0.082	0.145	1 (1.1)	0.03	0.03	0.03	0.03	0.03	0.199
	AFM_1	2 (1.1)	0.003	0.003	0.006	0.009	0.009	1 (1.1)	0.003	0.003	0.003	0.003	0.003	1 (1.1)	0.009	0.009	0.009	0.009	0.009	1
	AFB_1	0	ND	ND	ND	ND	ND	0	ND	ND	ND	ND	ND	0	ND	ND	ND	ND	ND	/
	AFM_2	0	ND	ND	ND	ND	ND	0	ND	ND	ND	ND	ND	0	ND	ND	ND	ND	ND	/
Ochratoxins	Total	184 (100.0)	0.131	0.61	0.989	1.709	16.047	89 (100.0)	0.19	0.706	1.154	2.048	16.05	95 (100.0)	0.131	0.587	0.862	1.398	6.409	/
	OTA	181 (98.4)	0.117	0.56	0.904	1.57	14.103	86 (96.6)	0.18	0.67	1.07	1.93	14.1	95 (100.0)	0.12	0.53	0.76	1.296	5.39	0.111
	OTB	184 (100.0)	0.008	0.045	0.076	0.16	1.947	89 (100.0)	0.01	0.052	0.095	0.167	1.947	95 (100.0)	0.008	0.041	0.066	0.129	1.89	/
	OTα	0	ND	ND	ND	ND	ND	0	ND	ND	ND	ND	ND	0	ND	ND	ND	ND	ND	/
Fumonisins	Total	82 (44.6)	0.02	0.131	0.212	0.358	1.227	45 (50.6)	0.024	0.121	0.2	0.306	0.574	37 (38.9)	0.02	0.151	0.254	0.365	1.227	0.113
	FB_1	14 (7.6)	0.131	0.194	0.318	0.473	0.818	7 (7.9)	0.131	0.167	0.217	0.567	0.574	7 (7.4)	0.194	0.249	0.332	0.37	0.818	0.899
	FB_2	45 (24.5)	0.041	0.18	0.23	0.283	0.665	25 (28.1)	0.041	0.176	0.229	0.283	0.454	20 (21.1)	0.158	0.183	0.253	0.292	0.665	0.267
	FB_3	38 (20.7)	0.02	0.052	0.101	0.156	0.695	17 (19.1)	0.024	0.056	0.097	0.138	0.368	21 (22.1)	0.02	0.044	0.104	0.162	0.695	0.615
T2 & HT2	Total	2 (1.1)	0.007	0.007	0.008	0.009	0.009	2 (2.3)	0.007	0.007	0.008	0.009	0.009	0 (0.0)	ND	ND				
	T2	2 (1.1)	0.007	0.007	0.008	0.009	0.009	2 (2.3)	0.007	0.007	0.008	0.009	0.009	0 (0.0)	ND	ND	ND	ND	ND	0.233
	HT2	0	ND	ND	ND	ND	ND	0	ND	ND	ND	ND	ND	0	ND	ND	ND	ND	ND	/
	T2(OH)3	0	ND	ND	ND	ND	ND	0	ND	ND	ND	ND	ND	0	ND	ND	ND	ND	ND	/
DON and its derivatives	Total	33 (17.9)	0.077	0.136	0.173	0.26	0.711	20 (22.5)	0.077	0.124	0.165	0.286	0.711	13 (13.7)	0.077	0.147	0.207	0.244	0.333	0.12
	3AcDON	3 (1.6)	0.077	0.077	0.104	0.104	0.104	3 (3.4)	0.077	0.077	0.104	0.104	0.104	0 (0.0)	ND	ND	ND	ND	ND	0.111
	DON	6 (3.3)	0.201	0.231	0.249	0.273	0.399	1 (1.1)	0.399	0.399	0.399	0.399	0.399	5 (5.3)	0.201	0.231	0.244	0.255	0.273	0.213
	FusX	25 (13.6)	0.077	0.136	0.164	0.213	0.711	17 (19.1)	0.082	0.137	0.166	0.26	0.711	8 (8.4)	0.077	0.115	0.155	0.21	0.333	0.035
	NIV	0	ND	ND	ND	ND	ND	0	ND	ND	ND	ND	ND	0	ND	ND	ND	ND	ND	/
	15AcDON	0	ND	ND	ND	ND	ND	0	ND	ND	ND	ND	ND	0	ND	ND	ND	ND	ND	/
	DOM	0	ND	ND	ND	ND	ND	0	ND	ND	ND	ND	ND	0	ND	ND	ND	ND	ND	/

	D3G	0	ND	ND	ND	ND	ND	0	ND	ND	ND	ND	ND	0	ND	ND	ND	ND	ND	/
	Total	21 (11.4)	0.005	0.008	0.031	0.062	3.194	13 (14.6)	0.005	0.008	0.029	0.053	0.273	8 (8.4)	0.005	0.011	0.06	0.114	3.194	0.187
	α-ZAL	3 (1.6)	0.053	0.053	0.057	0.27	0.27	2 (2.3)	0.053	0.053	0.162	0.27	0.27	1 (1.1)	0.057	0.057	0.057	0.057	0.057	0.611
	α-ZEL	1 (0.5)	0.041	0.041	0.041	0.041	0.041	1 (1.1)	0.041	0.041	0.041	0.041	0.041	0 (0.0)	ND	ND	ND	ND	ND	0.484
ZEN and its derivatives	ZEN	17 (9.2)	0.005	0.008	0.013	0.062	3.194	10 (11.2)	0.005	0.007	0.009	0.031	0.273	7 (7.4)	0.005	0.009	0.062	0.165	3.194	0.365
	ZAN	0	ND	ND	ND	ND	ND	0	ND	ND	ND	ND	ND	0	ND	ND	ND	ND	ND	/
	β-ZAL	0	ND	ND	ND	ND	ND	0	ND	ND	ND	ND	ND	0	ND	ND	ND	ND	ND	/
	β-ZEL	0	ND	ND	ND	ND	ND	0	ND	ND	ND	ND	ND	0	ND	ND	ND	ND	ND	/
∑ traditional mycotoxins			0.243	0.906	1.432	2.428	21.333		0.306	0.981	1.576	2.733	17.762		0.239	0.907	1.4	2.151	11.211	
**Emerging mycotoxins**																				
	Total	165 (89.7)	0.002	0.093	0.112	0.128	0.338	80 (89.9)	0.002	0.07	0.112	0.129	0.179	85 (89.5)	0.002	0.093	0.112	0.125	0.338	0.927
	BEA	126 (68.5)	0.087	0.104	0.117	0.126	0.174	60 (67.4)	0.095	0.107	0.116	0.131	0.174	66 (69.5)	0.087	0.103	0.117	0.124	0.172	0.764
Cyclohexaester peptide	EnA_1	5 (2.7)	0.002	0.002	0.003	0.003	0.007	4 (4.5)	0.002	0.002	0.003	0.003	0.003	1 (1.1)	0.007	0.007	0.007	0.007	0.007	0.199
	EnB	142 (77.2)	0.002	0.003	0.004	0.006	0.147	68 (76.4)	0.002	0.002	0.004	0.005	0.037	74 (77.9)	0.002	0.003	0.004	0.006	0.147	0.81
	EnB_1	18 (9.8)	0.002	0.003	0.003	0.004	0.012	8 (9.0)	0.003	0.003	0.003	0.004	0.006	10 (10.5)	0.002	0.002	0.003	0.003	0.012	0.726
	EnA	0	ND	ND	ND	ND	ND	0	ND	ND	ND	ND	ND	0	ND	ND	ND	ND	ND	/
	Total	22 (12.0)	0.003	0.011	0.026	0.031	0.054	12 (13.5)	0.008	0.024	0.031	0.036	0.054	10 (10.5)	0.003	0.005	0.016	0.025	0.049	0.537
	TEN	13 (7.1)	0.02	0.023	0.031	0.038	0.054	8 (9.0)	0.02	0.031	0.032	0.045	0.054	5 (5.3)	0.021	0.022	0.023	0.027	0.049	0.499
	AME	9 (4.9)	0.003	0.003	0.005	0.008	0.031	3 (3.4)	0.003	0.003	0.008	0.031	0.031	6 (6.3)	0.003	0.003	0.005	0.008	0.011	0.484
Alternaria toxins	AOH	1 (0.5)	0.025	0.025	0.025	0.025	0.025	1 (1.1)	0.025	0.025	0.025	0.025	0.025	0 (0.0)	ND	ND	ND	ND	ND	0.484
	ALT	0	ND	ND	ND	ND	ND	0	ND	ND	ND	ND	ND	0	ND	ND	ND	ND	ND	/
	TeA	0	ND	ND	ND	ND	ND	0	ND	ND	ND	ND	ND	0	ND	ND	ND	ND	ND	/
	AXT I	0	ND	ND	ND	ND	ND	0	ND	ND	ND	ND	ND	0	ND	ND	ND	ND	ND	/
	Total	93 (50.5)	0.001	0.002	0.003	0.014	0.059	52 (58.4)	0.001	0.002	0.003	0.014	0.059	41 (43.2)	0.001	0.002	0.003	0.014	0.051	0.038
	Acl	26 (14.1)	0.002	0.003	0.003	0.004	0.006	14 (15.7)	0.003	0.003	0.003	0.004	0.006	12 (12.6)	0.002	0.003	0.003	0.004	0.004	0.546
	DH-Ecr	1 (0.5)	0.021	0.021	0.021	0.021	0.021	1 (1.1)	0.021	0.021	0.021	0.021	0.021	0 (0.0)	ND	ND	ND	ND	ND	0.484
Ergot alkaloids	DH-LYS	25 (13.6)	0.011	0.014	0.017	0.028	0.059	11 (12.4)	0.013	0.016	0.02	0.031	0.059	14 (14.7)	0.011	0.014	0.016	0.027	0.051	0.638
	Econ	3 (1.6)	0.002	0.002	0.002	0.003	0.003	1 (1.1)	0.002	0.002	0.002	0.002	0.002	2 (2.1)	0.002	0.002	0.002	0.003	0.003	1
	Ecrn	3 (1.6)	0.013	0.013	0.014	0.015	0.015	3 (3.4)	0.013	0.013	0.014	0.015	0.015	0 (0.0)	ND	ND	ND	ND	ND	0.111
	Em	2 (1.1)	0.001	0.001	0.001	0.002	0.002	2 (2.3)	0.001	0.001	0.001	0.002	0.002	0 (0.0)	ND	ND	ND	ND	ND	0.233
	Esn	44 (23.9)	0.001	0.001	0.002	0.002	0.003	30 (33.7)	0.001	0.001	0.001	0.002	0.003	14 (14.7)	0.001	0.002	0.002	0.002	0.002	0.003

	Ecl	0	ND	ND	ND	ND	ND	0	ND	ND	ND	ND	ND	0	ND	ND	ND	ND	ND	/
	Etn	0	ND	ND	ND	ND	ND	0	ND	ND	ND	ND	ND	0	ND	ND	ND	ND	ND	/
	Ergine	0	ND	ND	ND	ND	ND	0	ND	ND	ND	ND	ND	0	ND	ND	ND	ND	ND	/
	Eco	0	ND	ND	ND	ND	ND	0	ND	ND	ND	ND	ND	0	ND	ND	ND	ND	ND	/
	Ecr	0	ND	ND	ND	ND	ND	0	ND	ND	ND	ND	ND	0	ND	ND	ND	ND	ND	/
	Emn	0	ND	ND	ND	ND	ND	0	ND	ND	ND	ND	ND	0	ND	ND	ND	ND	ND	/
	Ek	0	ND	ND	ND	ND	ND	0	ND	ND	ND	ND	ND	0	ND	ND	ND	ND	ND	/
	Ekn	0	ND	ND	ND	ND	ND	0	ND	ND	ND	ND	ND	0	ND	ND	ND	ND	ND	/
	Et	0	ND	ND	ND	ND	ND	0	ND	ND	ND	ND	ND	0	ND	ND	ND	ND	ND	/
Diacetoxyscirpenol	Total	14 (7.6)	0.005	0.016	0.029	0.123	0.222	6 (6.7)	0.013	0.016	0.022	0.027	0.161	8 (8.4)	0.005	0.021	0.077	0.142	0.222	0.668
	15AS	12 (6.5)	0.013	0.016	0.023	0.031	0.161	6 (6.7)	0.013	0.016	0.022	0.027	0.161	6 (6.3)	0.013	0.015	0.025	0.032	0.121	0.907
	DAS	3 (1.6)	0.103	0.103	0.145	0.222	0.222	0 (0.0)	ND	ND	ND	ND	ND	3 (3.2)	0.103	0.103	0.145	0.222	0.222	0.247
	NEO	1 (0.5)	0.005	0.005	0.005	0.005	0.005	0 (0.0)	ND	ND	ND	ND	ND	1 (1.1)	0.005	0.005	0.005	0.005	0.005	1
Penicillin	Total	147 (79.9)	0.084	0.158	0.288	0.575	11.137	72 (80.9)	0.1	0.245	0.333	0.78	11.137	75 (79.0)	0.084	0.136	0.198	0.424	2.323	0.741
	CIT	146 (79.3)	0.084	0.158	0.288	0.573	7.794	71 (79.8)	0.1	0.244	0.329	0.6	7.794	75 (79.0)	0.084	0.136	0.198	0.424	2.323	0.89
	PAT	2 (1.1)	4.639	4.639	7.522	10.41	10.41	2 (2.3)	4.639	4.639	7.522	10.41	10.41	0 (0.0)	ND	ND	ND	ND	ND	0.233
	PCA	3 (1.6)	0.046	0.046	0.052	0.163	0.163	3 (3.4)	0.046	0.046	0.052	0.163	0.163	0 (0.0)	ND	ND	ND	ND	ND	0.111
	RC	0	ND	ND	ND	ND	ND	0	ND	ND	ND	ND	ND	0	ND	ND	ND	ND	ND	/
other mycotoxins	CPA	82 (44.6)	0.001	0.001	0.002	0.003	1.363	42 (47.2)	0.001	0.001	0.002	0.003	1.363	40 (42.1)	0.001	0.001	0.002	0.003	0.018	0.488
	GLIO	3 (1.6)	0.105	0.105	0.107	0.116	0.116	1 (1.1)	0.107	0.107	0.107	0.107	0.107	2 (2.1)	0.105	0.105	0.11	0.116	0.116	1
	MPA	9 (4.9)	0.057	0.096	0.109	0.123	0.236	8 (9.0)	0.057	0.081	0.104	0.119	0.236	1 (1.1)	0.204	0.204	0.204	0.204	0.204	0.016
	STG	1 (0.5)	0.001	0.001	0.001	0.001	0.001	1 (1.1)	0.001	0.001	0.001	0.001	0.001	0 (0.0)	ND	ND	ND	ND	ND	0.484
	MON	0	ND	ND	ND	ND	ND	0	ND	ND	ND	ND	ND	0	ND	ND	ND	ND	ND	/
∑ emerging mycotoxins			0.259	0.483	0.677	1.114	13.526		0.29	0.547	0.715	1.216	13.297		0.405	0.567	0.722	1.053	3.321	
∑ mycotoxins			0.502	1.389	2.109	3.542	34.859		0.596	1.528	2.291	3.949	31.059		0.644	1.474	2.122	3.204	14.532	

ND: level below LOD; *Chi-squared or Fisher's exact test was performed to compare the difference of detection rate between the case and control groups.

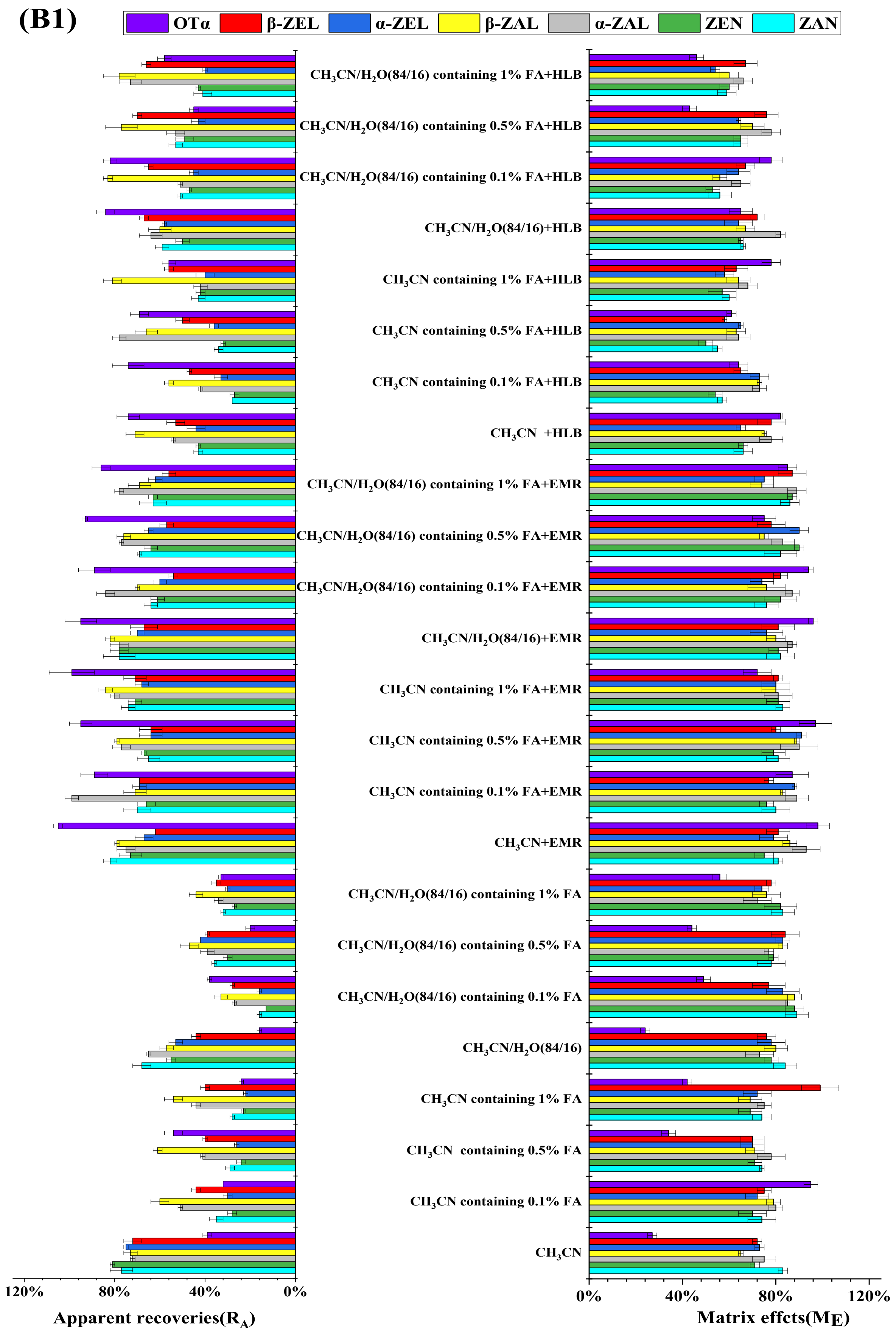
(B1)
OTα
β-ZEL
α-ZEL
β-ZAL
α-ZAL
ZEN
ZAN
CH3CN/H2O(84/16) containing 1% FA+HLB
CH3CN/H2O(84/16) containing 0.5% FA+HLB
CH3CN/H2O(84/16) containing 0.1% FA+HLB
CH3CN/H2O(84/16)+HLB
CH3CN containing 1% FA+HLB
CH3CN containing 0.5% FA+HLB
CH3CN containing 0.1% FA+HLB
CH3CN +HLB
CH3CN/H2O(84/16) containing 1% FA+EMR
CH3CN/H2O(84/16) containing 0.5% FA+EMR
CH3CN/H2O(84/16) containing 0.1% FA+EMR
CH3CN/H2O(84/16)+EMR
CH3CN containing 1% FA+EMR
CH3CN containing 0.5% FA+EMR
CH3CN containing 0.1% FA+EMR
CH3CN+EMR
CH3CN/H2O(84/16) containing 1% FA
CH3CN/H2O(84/16) containing 0.5% FA
CH3CN/H2O(84/16) containing 0.1% FA
CH3CN/H2O(84/16)
CH3CN containing 1% FA
CH3CN containing 0.5% FA
CH3CN containing 0.1% FA
CH3CN
120%
80%
40%
0%
Apparent recoveries(RA)
0%
40%
80%
120%
Matrix effcts(ME)

Figure S1

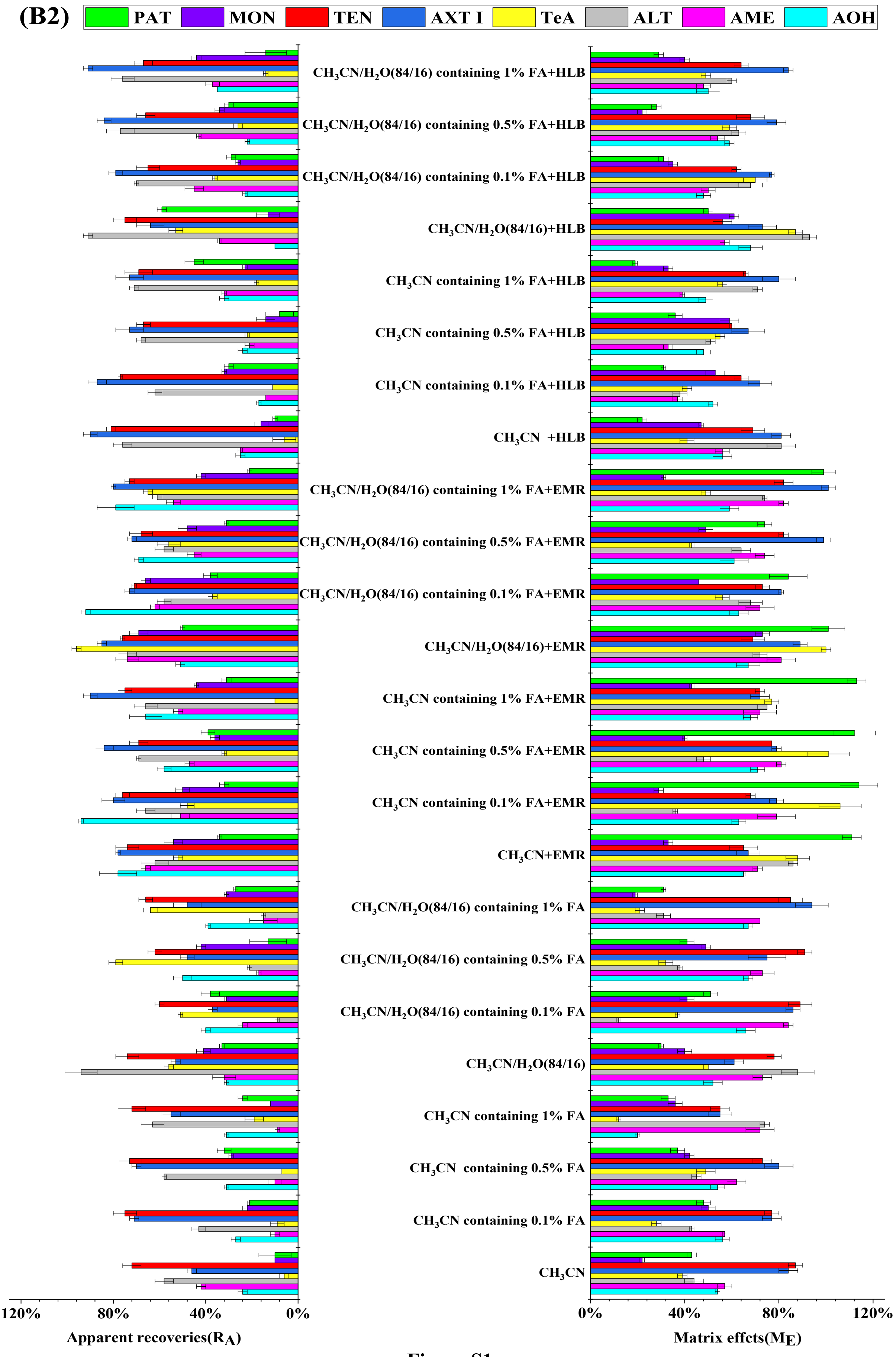
(B2)
PAT
MON
TEN
AXT I
TeA
ALT
AME
AOH
CH3CN/H2O(84/16) containing 1% FA+HLB
CH3CN/H2O(84/16) containing 0.5% FA+HLB
CH3CN/H2O(84/16) containing 0.1% FA+HLB
CH3CN/H2O(84/16)+HLB
CH3CN containing 1% FA+HLB
CH3CN containing 0.5% FA+HLB
CH3CN containing 0.1% FA+HLB
CH3CN +HLB
CH3CN/H2O(84/16) containing 1% FA+EMR
CH3CN/H2O(84/16) containing 0.5% FA+EMR
CH3CN/H2O(84/16) containing 0.1% FA+EMR
CH3CN/H2O(84/16)+EMR
CH3CN containing 1% FA+EMR
CH3CN containing 0.5% FA+EMR
CH3CN containing 0.1% FA+EMR
CH3CN+EMR
CH3CN/H2O(84/16) containing 1% FA
CH3CN/H2O(84/16) containing 0.5% FA
CH3CN/H2O(84/16) containing 0.1% FA
CH3CN/H2O(84/16)
CH3CN containing 1% FA
CH3CN containing 0.5% FA
CH3CN containing 0.1% FA
CH3CN
120%
80%
40%
0%
0%
40%
80%
120%
Apparent recoveries(RA)
Matrix effcts(ME)

Figure S1

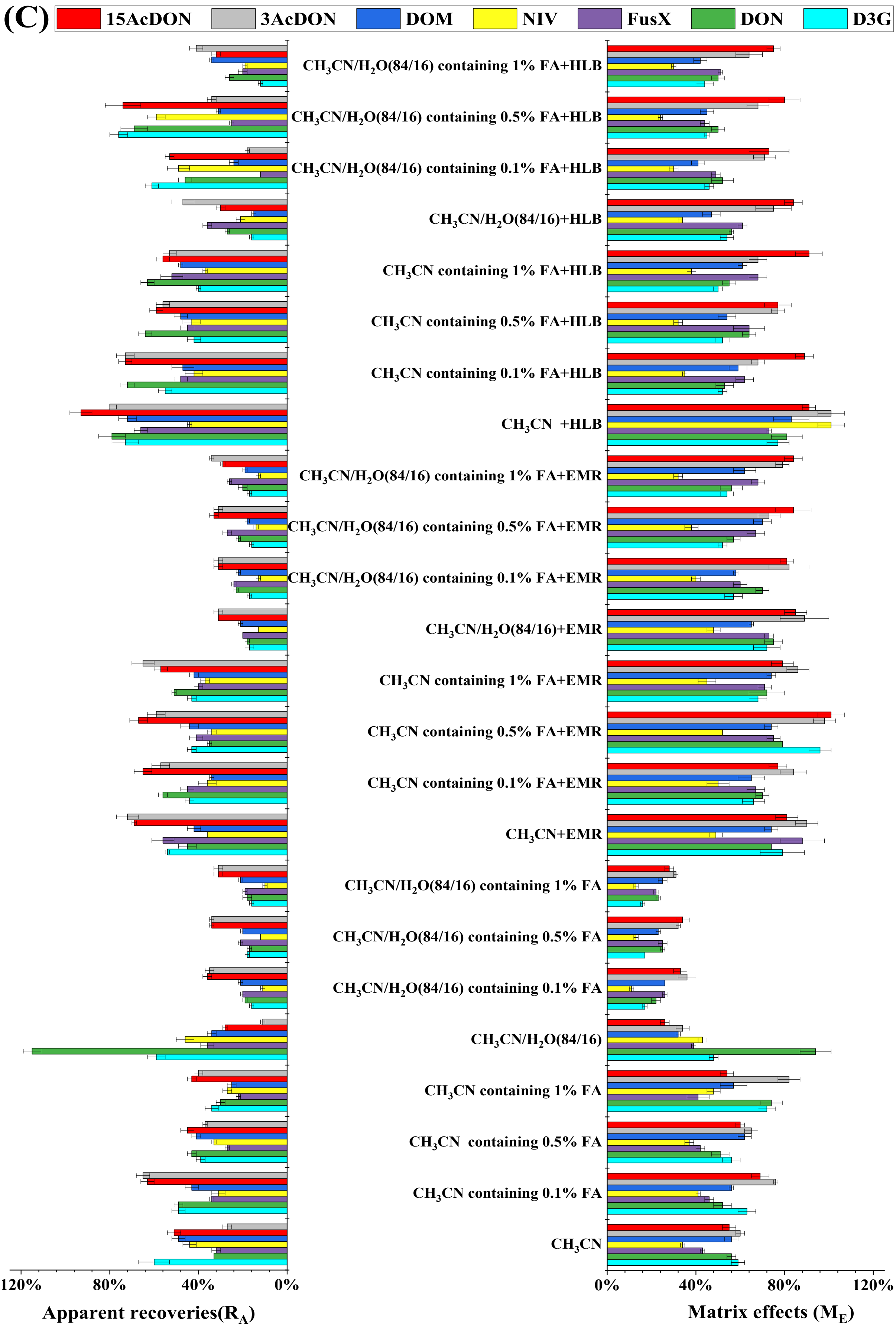
(C)
15AcDON
3AcDON
DOM
NIV
FusX
DON
D3G
CH3CN/H2O(84/16) containing 1% FA+HLB
CH3CN/H2O(84/16) containing 0.5% FA+HLB
CH3CN/H2O(84/16) containing 0.1% FA+HLB
CH3CN/H2O(84/16)+HLB
CH3CN containing 1% FA+HLB
CH3CN containing 0.5% FA+HLB
CH3CN containing 0.1% FA+HLB
CH3CN +HLB
CH3CN/H2O(84/16) containing 1% FA+EMR
CH3CN/H2O(84/16) containing 0.5% FA+EMR
CH3CN/H2O(84/16) containing 0.1% FA+EMR
CH3CN/H2O(84/16)+EMR
CH3CN containing 1% FA+EMR
CH3CN containing 0.5% FA+EMR
CH3CN containing 0.1% FA+EMR
CH3CN+EMR
CH3CN/H2O(84/16) containing 1% FA
CH3CN/H2O(84/16) containing 0.5% FA
CH3CN/H2O(84/16) containing 0.1% FA
CH3CN/H2O(84/16)
CH3CN containing 1% FA
CH3CN containing 0.5% FA
CH3CN containing 0.1% FA
CH3CN
120%
80%
40%
0%
0%
40%
80%
120%
Apparent recoveries(RA)
Matrix effects (ME)

Figure S1

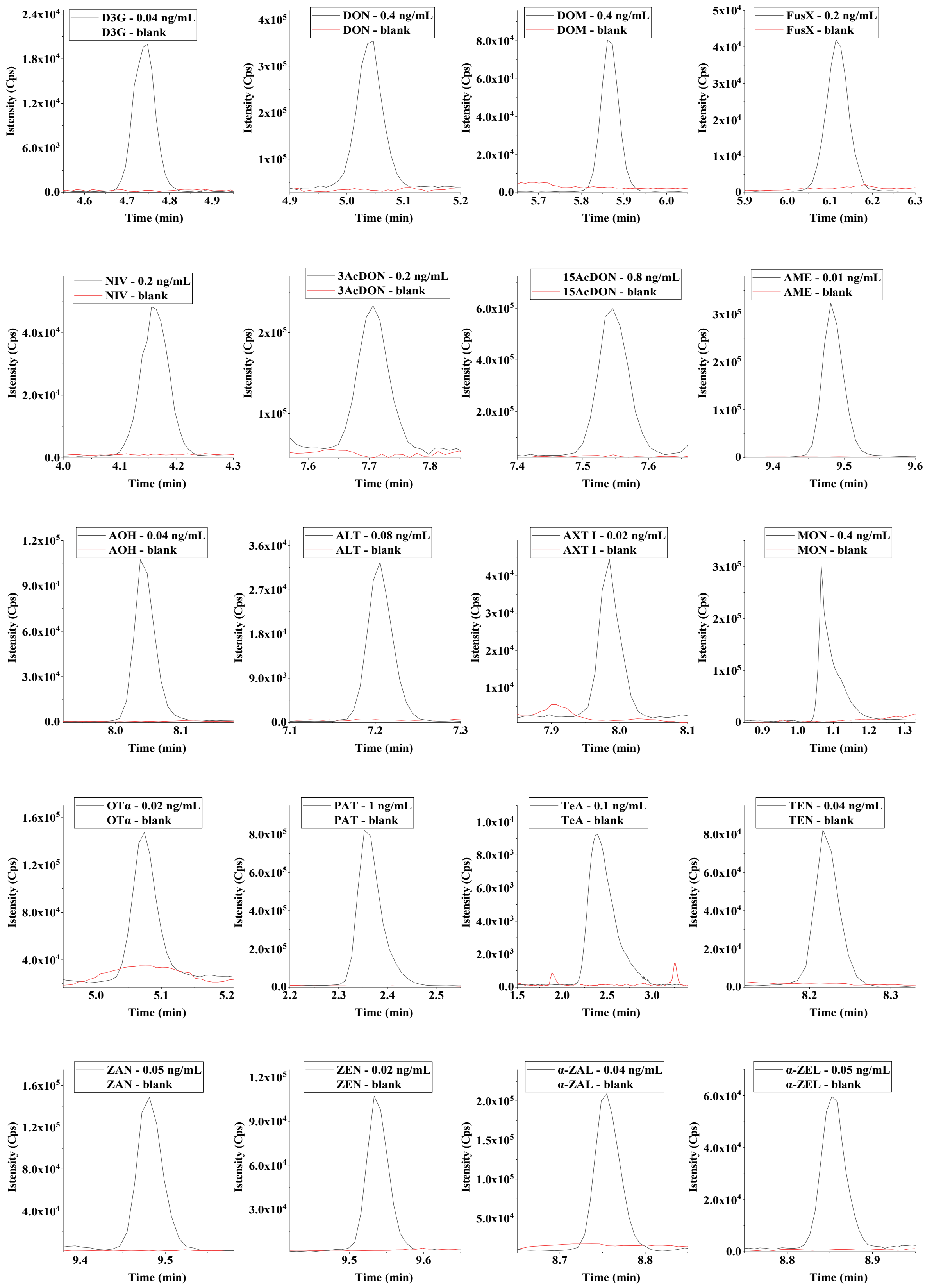
D3G - 0.04 ng/mL
D3G - blank
DON - 0.4 ng/mL
DON - blank
DOM - 0.4 ng/mL
DOM - blank
FusX - 0.2 ng/mL
FusX - blank
NIV - 0.2 ng/mL
NIV - blank
3AcDON - 0.2 ng/mL
3AcDON - blank
15AcDON - 0.8 ng/mL
15AcDON - blank
AME - 0.01 ng/mL
AME - blank
AOH - 0.04 ng/mL
AOH - blank
ALT - 0.08 ng/mL
ALT - blank
AXT I - 0.02 ng/mL
AXT I - blank
MON - 0.4 ng/mL
MON - blank
OTα - 0.02 ng/mL
OTα - blank
PAT - 1 ng/mL
PAT - blank
TeA - 0.1 ng/mL
TeA - blank
TEN - 0.04 ng/mL
TEN - blank
ZAN - 0.05 ng/mL
ZAN - blank
ZEN - 0.02 ng/mL
ZEN - blank
α-ZAL - 0.04 ng/mL
α-ZAL - blank
α-ZEL - 0.05 ng/mL
α-ZEL - blank
Istensity (Cps)
Time (min)

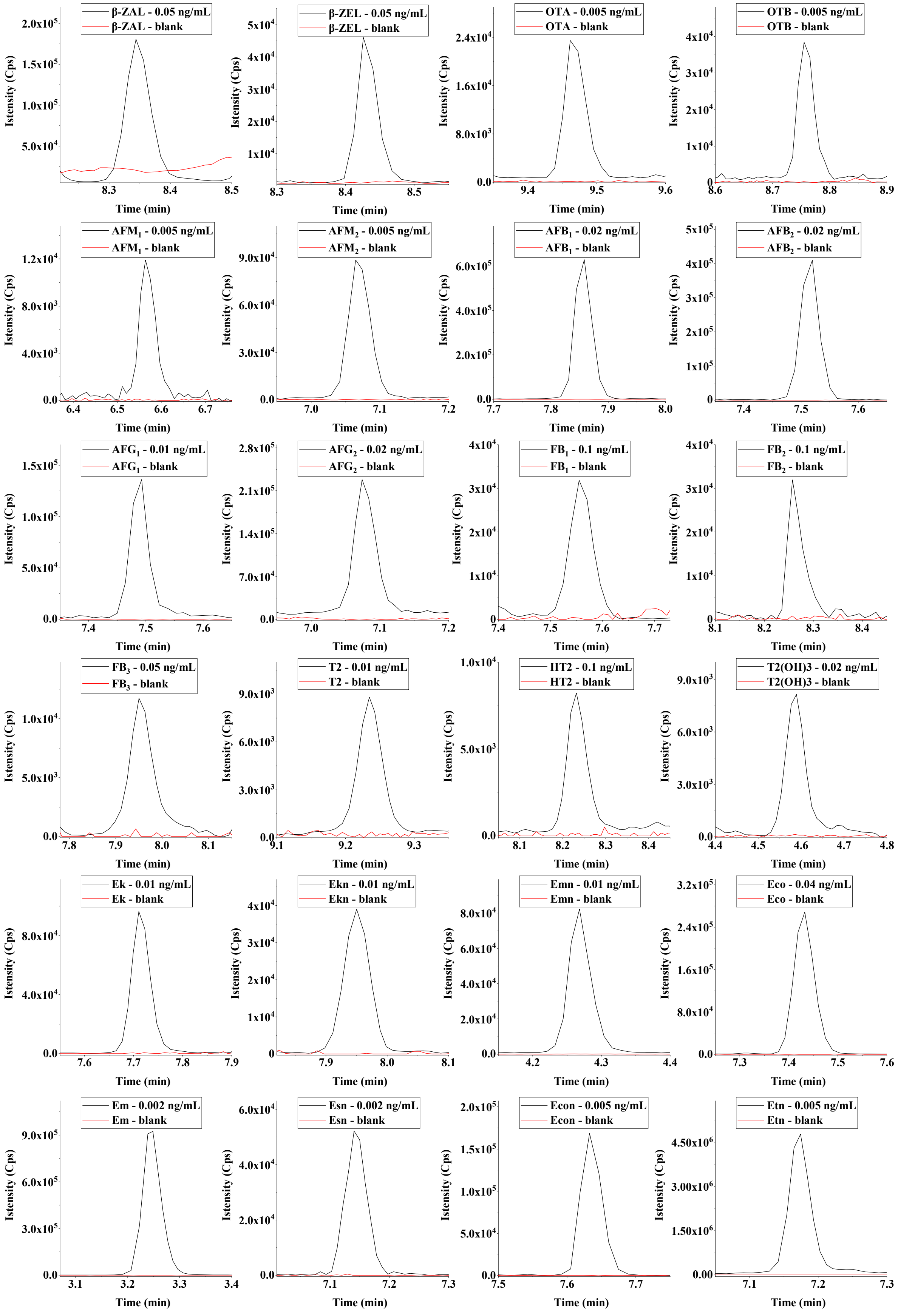
β-ZAL - 0.05 ng/mL
β-ZAL - blank
β-ZEL - 0.05 ng/mL
β-ZEL - blank
OTA - 0.005 ng/mL
OTA - blank
OTB - 0.005 ng/mL
OTB - blank
AFM$_1$ - 0.005 ng/mL
AFM$_1$ - blank
AFM$_2$ - 0.005 ng/mL
AFM$_2$ - blank
AFB$_1$ - 0.02 ng/mL
AFB$_1$ - blank
AFB$_2$ - 0.02 ng/mL
AFB$_2$ - blank
AFG$_1$ - 0.01 ng/mL
AFG$_1$ - blank
AFG$_2$ - 0.02 ng/mL
AFG$_2$ - blank
FB$_1$ - 0.1 ng/mL
FB$_1$ - blank
FB$_2$ - 0.1 ng/mL
FB$_2$ - blank
FB$_3$ - 0.05 ng/mL
FB$_3$ - blank
T2 - 0.01 ng/mL
T2 - blank
HT2 - 0.1 ng/mL
HT2 - blank
T2(OH)3 - 0.02 ng/mL
T2(OH)3 - blank
Ek - 0.01 ng/mL
Ek - blank
Ekn - 0.01 ng/mL
Ekn - blank
Emn - 0.01 ng/mL
Emn - blank
Eco - 0.04 ng/mL
Eco - blank
Em - 0.002 ng/mL
Em - blank
Esn - 0.002 ng/mL
Esn - blank
Econ - 0.005 ng/mL
Econ - blank
Etn - 0.005 ng/mL
Etn - blank
Istensity (Cps)
Time (min)

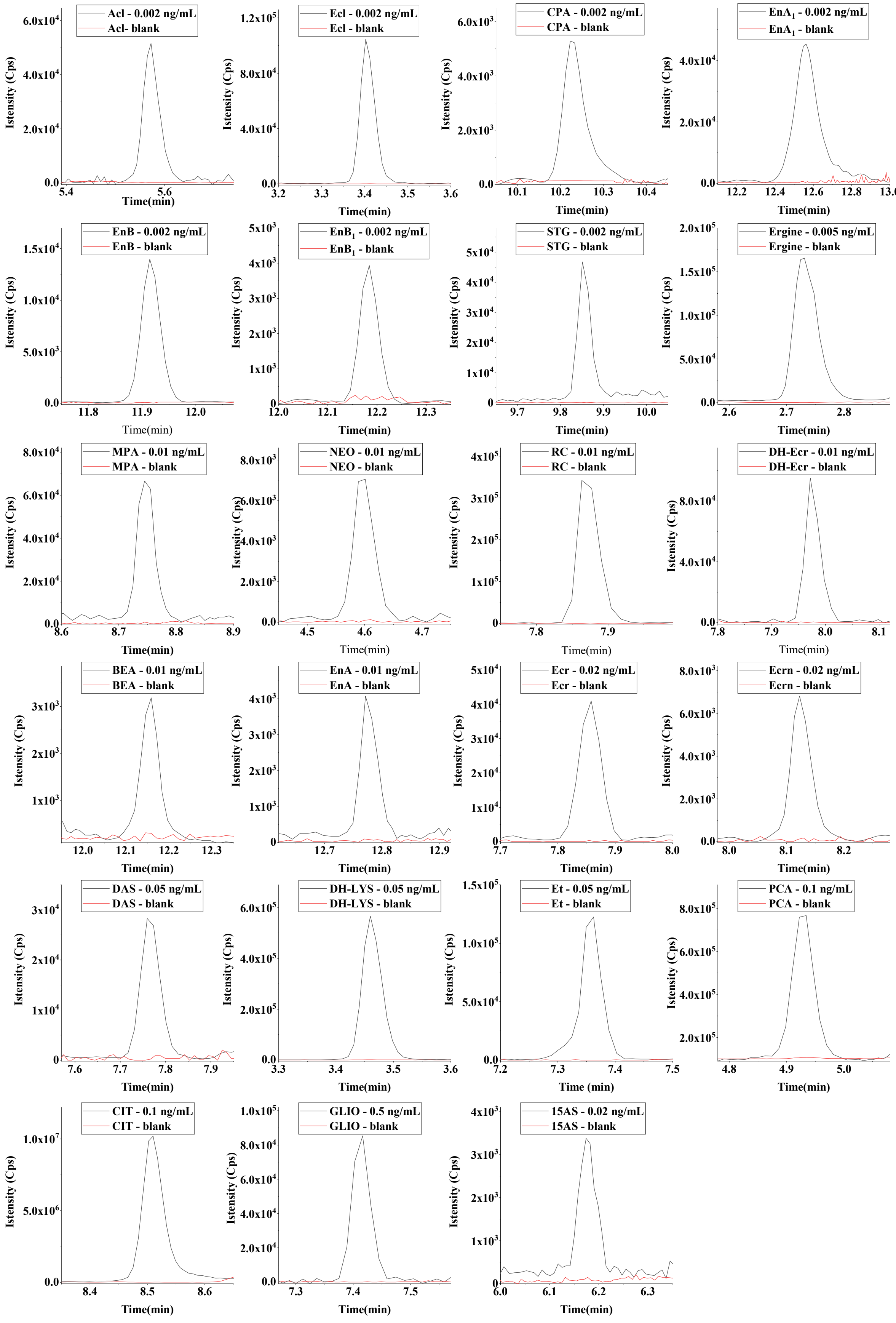
Acl - 0.002 ng/mL
Acl- blank
Ecl - 0.002 ng/mL
Ecl - blank
CPA - 0.002 ng/mL
CPA - blank
EnA1 - 0.002 ng/mL
EnA1 - blank
EnB - 0.002 ng/mL
EnB - blank
EnB1 - 0.002 ng/mL
EnB1 - blank
STG - 0.002 ng/mL
STG - blank
Ergine - 0.005 ng/mL
Ergine - blank
MPA - 0.01 ng/mL
MPA - blank
NEO - 0.01 ng/mL
NEO - blank
RC - 0.01 ng/mL
RC - blank
DH-Ecr - 0.01 ng/mL
DH-Ecr - blank
BEA - 0.01 ng/mL
BEA - blank
EnA - 0.01 ng/mL
EnA - blank
Ecr - 0.02 ng/mL
Ecr - blank
Ecrn - 0.02 ng/mL
Ecrn - blank
DAS - 0.05 ng/mL
DAS - blank
DH-LYS - 0.05 ng/mL
DH-LYS - blank
Et - 0.05 ng/mL
Et - blank
PCA - 0.1 ng/mL
PCA - blank
CIT - 0.1 ng/mL
CIT - blank
GLIO - 0.5 ng/mL
GLIO - blank
15AS - 0.02 ng/mL
15AS - blank
Istensity (Cps)
Time(min)

Figure S2

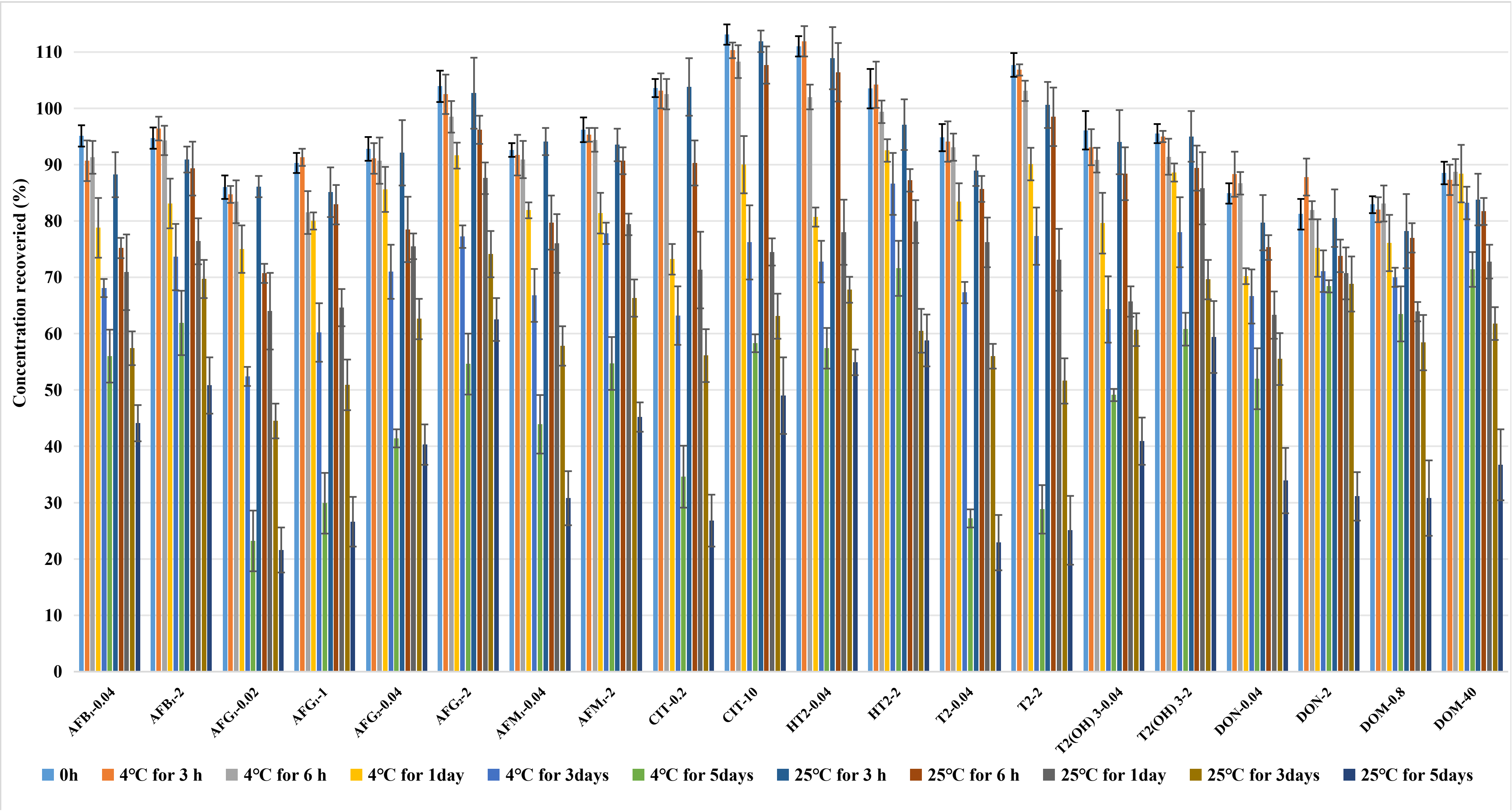

Concentration recoveried (%)
0
10
20
30
40
50
60
70
80
90
100
110
AFB$_1$-0.04
AFB$_1$-2
AFG$_1$-0.02
AFG$_1$-1
AFG$_2$-0.04
AFG$_2$-2
AFM$_1$-0.04
AFM$_1$-2
CIT-0.2
CIT-10
HT2-0.04
HT2-2
T2-0.04
T2-2
T2(OH) 3-0.04
T2(OH) 3-2
DON-0.04
DON-2
DOM-0.8
DOM-40
0h
4°C for 3 h
4°C for 6 h
4°C for 1day
4°C for 3days
4°C for 5days
25°C for 3 h
25°C for 6 h
25°C for 1day
25°C for 3days
25°C for 5days

Figure S3

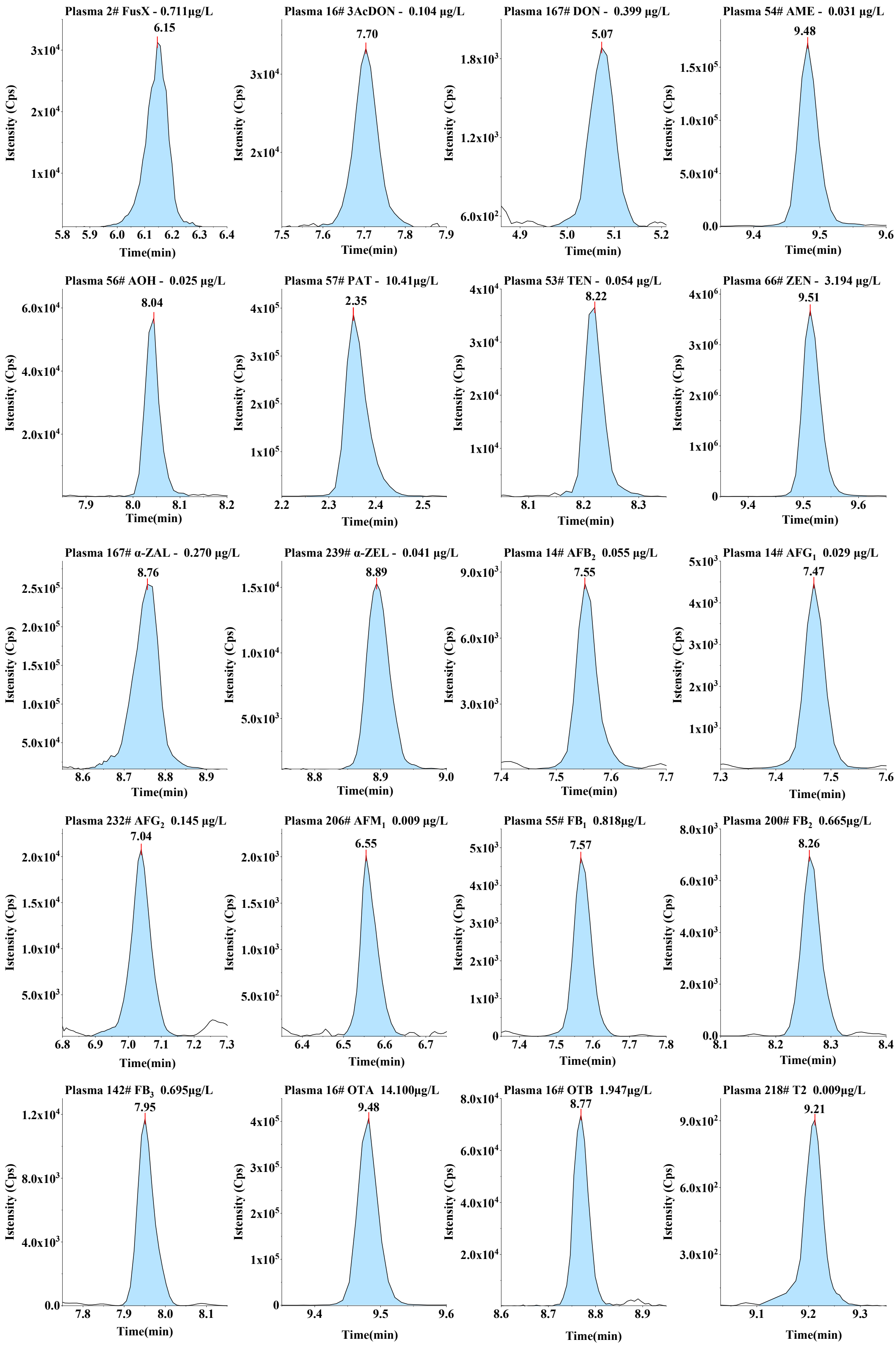

Plasma 2# FusX - 0.711μg/L
6.15
Plasma 16# 3AcDON - 0.104 μg/L
7.70
Plasma 167# DON - 0.399 μg/L
5.07
Plasma 54# AME - 0.031 μg/L
9.48
Plasma 56# AOH - 0.025 μg/L
8.04
Plasma 57# PAT - 10.41μg/L
2.35
Plasma 53# TEN - 0.054 μg/L
8.22
Plasma 66# ZEN - 3.194 μg/L
9.51
Plasma 167# α-ZAL - 0.270 μg/L
8.76
Plasma 239# α-ZEL - 0.041 μg/L
8.89
Plasma 14# AFB_2 0.055 μg/L
7.55
Plasma 14# AFG_1 0.029 μg/L
7.47
Plasma 232# AFG_2 0.145 μg/L
7.04
Plasma 206# AFM_1 0.009 μg/L
6.55
Plasma 55# FB_1 0.818μg/L
7.57
Plasma 200# FB_2 0.665μg/L
8.26
Plasma 142# FB_3 0.695μg/L
7.95
Plasma 16# OTA 14.100μg/L
9.48
Plasma 16# OTB 1.947μg/L
8.77
Plasma 218# T2 0.009μg/L
9.21
Istensity (Cps)
Time(min)

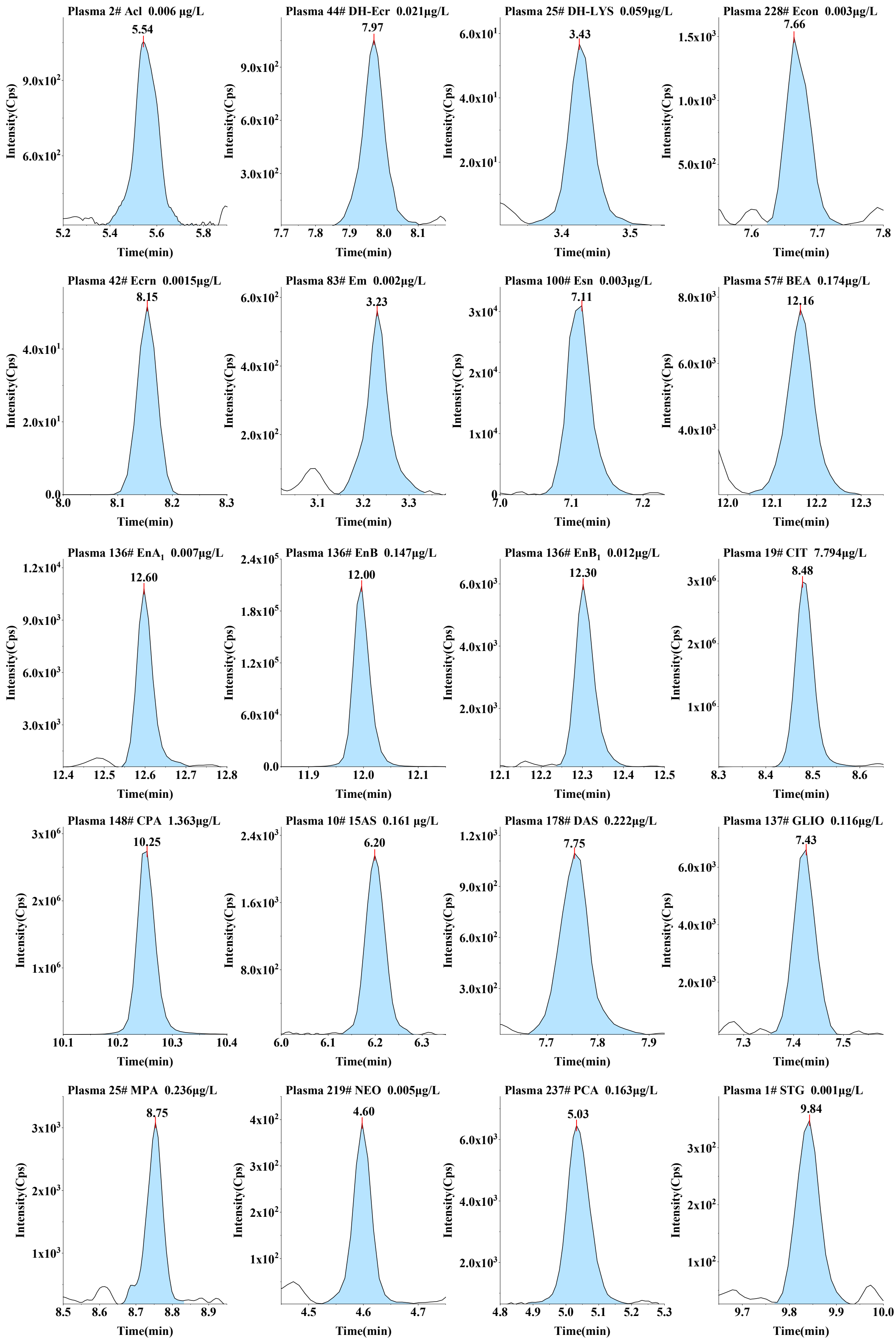
Plasma 2# Acl 0.006 μg/L
5.54
Plasma 44# DH-Ecr 0.021μg/L
7.97
Plasma 25# DH-LYS 0.059μg/L
3.43
Plasma 228# Econ 0.003μg/L
7.66
Plasma 42# Ecrn 0.0015μg/L
8.15
Plasma 83# Em 0.002μg/L
3.23
Plasma 100# Esn 0.003μg/L
7.11
Plasma 57# BEA 0.174μg/L
12.16
Plasma 136# EnA_1 0.007μg/L
12.60
Plasma 136# EnB 0.147μg/L
12.00
Plasma 136# EnB_1 0.012μg/L
12.30
Plasma 19# CIT 7.794μg/L
8.48
Plasma 148# CPA 1.363μg/L
10.25
Plasma 10# 15AS 0.161 μg/L
6.20
Plasma 178# DAS 0.222μg/L
7.75
Plasma 137# GLIO 0.116μg/L
7.43
Plasma 25# MPA 0.236μg/L
8.75
Plasma 219# NEO 0.005μg/L
4.60
Plasma 237# PCA 0.163μg/L
5.03
Plasma 1# STG 0.001μg/L
9.84
Intensity(Cps)
Time(min)

Figure S4
